# Electrospinning of Softwood Kraft Lignin With Cellulose Acetate: Dye Adsorption, Carbonization, and Carbon Dioxide Capture

**DOI:** 10.1002/cssc.70717

**Published:** 2026-05-15

**Authors:** Unnimaya Thalakkale Veettil, Fengyang Wang, Mirva Eriksson, Aleksander Jaworski, Mika H. Sipponen

**Affiliations:** ^1^ Department of Chemistry Stockholm University Stockholm Sweden

**Keywords:** carbonization, CO_2_ sorption, dye adsorption, electrospinning, softwood kraft lignin

## Abstract

The development of bio‐based materials capable of addressing multiple environmental challenges is critical for advancing sustainable materials design. Herein, we report electrospun nanofibers from unfractionated, chemically unmodified softwood kraft lignin and cellulose acetate, where cellulose acetate acts as a carrier polymer, enabling the formation of uniform precursor fibers with lignin contents of up to 80%. The resulting nanofibers enable sequential dye adsorption from water (methylene blue removal capacity of 19 mg/g) and carbon dioxide (CO_2_) capture (2.2 mmol/g) after carbonization. When evaluated solely for CO_2_ capture, the pristine carbonized materials show sorption capacities of 3.1–3.9 mmol/g, which increase as lignin content in the precursor fibers decreases, underscoring the importance of tuning carbon yield and capture performance. The sequential utilization of a single precursor material demonstrates a stepwise transformation that aligns with resource efficiency and sustainable material design. Overall, this study demonstrates a viable route for the direct valorization of industrial lignin into functional materials for both water purification and carbon capture applications.

## Graphical abstract

Electrospun nanofibers from unfractionated softwood kraft lignin and cellulose acetate enable up to 80% lignin incorporation without modification. The materials support sequential use, first for dye removal from water and subsequently for carbon dioxide capture after carbonization. Performance depends on lignin content, highlighting tunable structure–function relationships and the direct lignin valorization into multifunctional, resource‐efficient materials.

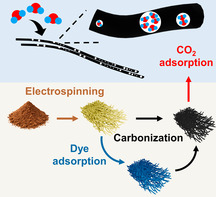

## 1 Introduction

The rapid expansion of industrial activities has intensified two interconnected challenges namely, water contamination and rising atmospheric carbon dioxide (CO_2_) levels, with the latter being the primary driver of climate change [1]. Mitigating CO_2_ emissions while enabling efficient remediation of industrial effluents is thus central to achieving climate neutrality and circular economy goals. Carbon capture technologies [2] and adsorption‐based separation processes have gained renewed attention, driven by the need for scalable, energy‐efficient, and sustainable material solutions. Conventional sorbent materials and treatment methods, while being effective, often suffer from limitations such as regeneration efficiency and reliance on nonrenewable resources [3]. Recent studies on adsorbent materials have focused on tailoring high surface‐area and chemical structures to enhance pollutant uptake [4], with increased emphasis on renewable feedstocks driving interest in bio‐derived alternatives.

Adsorption‐based separation has become a central strategy for addressing water pollution and carbon dioxide capture. A wide range of materials have been explored for gas and dye adsorption, including protein nanofibrils [5], metal–organic frameworks (MOFs) [6, 7, 8], porous organic polymers [9, 10, 11], ionic liquids [12], and carbon‐based materials [13], many of which show high adsorption capacities but rely on complex, energy‐intensive synthesis and non‐renewable precursors. For carbon capture, porous carbons remain among the most widely used sorbents because of their tunable porosity [14], chemical resistance [15], and thermal stability [16]. Simultaneously, increasing attention has turned to biomass‐derived carbons, particularly lignin, owing to its high aromatic carbon content and suitability for conversion into porous structures [17, 18]. Over the last decade, lignin has been explored in various forms including powders [19, 20], particles [21], fibers [22], and foams [23], which can potentially be used for the fabrication of carbon materials.

One of the emerging routes for lignin valorization is electrospinning [24]. Producing lignin fibers by electrospinning is challenging without chemical modification or solvent fractionation, due to the presence of low‐molecular‐weight fractions and the inherent heterogeneity of lignin [25, 26]. Though electrospinning of the mere technical lignin generally results in electrospraying of lignin particles [27], there have been multiple approaches to improve the spinnability by adding a binder polymer such as poly(ethylene oxide) (PEO) [28, 29], poly(acrylonitrile) (PAN) [30, 31], poly(lactic acid) (PLA) [32], poly(vinyl alcohol) (PVA) [33], cellulose acetate (CA) [27, 34, 35], or by solvent fractionation of lignin [36, 37]. Previous studies on electrospinning of lignin‐CA systems have primarily focused on hardwood kraft lignins [34]. Moreover, lignin‐based electrospun fibers have largely focused on single‐function applications such as water purification or carbonization for gas sorption or energy storage.

In the present work, we report the electrospinning of softwood kraft lignin (SKL) in the presence of CA, achieving precursor fiber mats with up to 80 wt.% lignin. Using CA as the sole binder polymer for electrospinning enabled a fully bio‐based fiber system while reducing the processing complexity associated with lignin fractionation or modification. The electrospun fibers were first evaluated for methylene blue adsorption from water and were subsequently carbonized for CO_2_ capture. In comparison, carbonization of pristine fibers without prior dye adsorption resulted in higher CO_2_ uptake than fibers carbonized after dye loading. These observations indicate that adsorbed dye interferes with pore development during carbonization. Overall, the results highlight the dual role of lignin in carbon fiber production: lignin is essential for achieving high carbon yields and structural integrity during thermal stabilization, while the surface area and micropore volume of the resulting carbon can be systematically tuned by adjusting the lignin content in the precursor fiber mats.

## 2 Results and Discussion

### 2.1 Optimization of Lignin Nanofiber Electrospinning

**FIGURE 1:**
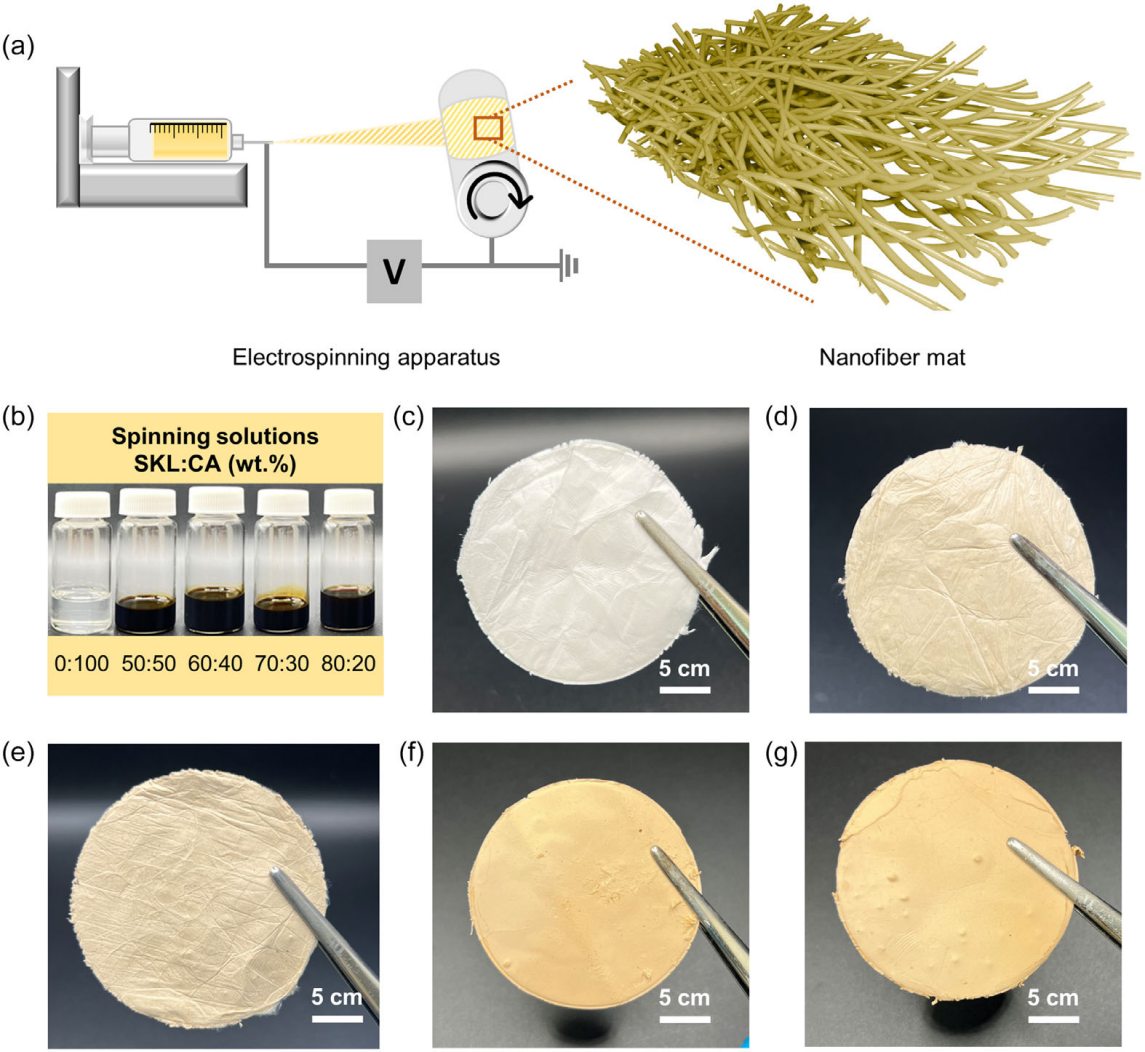
Electrospinning of lignin–cellulose acetate fibers. (a) Illustration of electrospinning setup with a rotating drum collector and the nanofibers formed. (b) Formulations of electrospinning solution used to spin the nanofibers (SKL–CA) shown in the digital images. (c) 0:100, (d) 50:50, (e) 60:40, (f) 70:30, and (g) 80:20 wt.%, respectively (electrospun fiber sheets were cut into circular pieces for representation only).

Electrospinning apparatus with a rotating drum collector, as shown in Figure 1a, was employed for efficient, long duration, and continuous spinning of SKL–CA solutions with different formulations. Electrospinning solutions were first obtained by dissolving SKL and CA in 0:100, 50:50, 60:40, 70:30, and 80:20 wt.%, respectively, in a 1:2.4 v/v ratio of DMF/acetone solvent mixture, as shown in Figure 1b. After several trial‐and‐error experiments, the values for the electrospinning parameters, such as flow rate, electrospinning voltage, distance between the nozzle and the collector as well as the speed of the rotating drum collector, were optimized. Figure 1c–g show the digital images of the nanofiber mats formed after 5–6 h of electrospinning at a flow rate of 0.03 mL/min, 20 kV voltage, 20 cm distance between the collector and the spinneret, and 130 rpm rotating drum speed. Similarly, an SKL–CA solution with a 100:0 wt.% composition primarily resulted in electrosprayed lignin particles rather than fibers.

Previous studies on lignin electrospinning have largely relied on blending lignin with a polymer to achieve sufficient spinnability. The lignin content in these prior studies has been up to 70% in the case of hardwood kraft lignin from poplar and eucalyptus. Alternatively, electrospinning of fractionated lignin without additives has been reported, yet these approaches rely on solvent‐intensive fractionation of lignin [36, 37]. Electrospinning of lignin without binder polymers has been demonstrated for softwood organosolv lignin; however, to suppress the bead formation and to improve the fiber uniformity, addition of iron (III) chloride was required [38]. In some systems, lignin's tendency to electrospray has been deliberately utilized to produce fiber bead structures using hardwood kraft lignin from eucalyptus/CA [27]. Since pure lignin lacks sufficient chain entanglement and viscoelasticity for fiber spinning, it is commonly blended with polymers such as PAN [30, 31, 39], PEO [28, 29], PLA [32], and CA [34], to improve the electrospinnability. These compositional and rheological factors influence the morphology of the resulting electrospun fibers.

**FIGURE 2:**
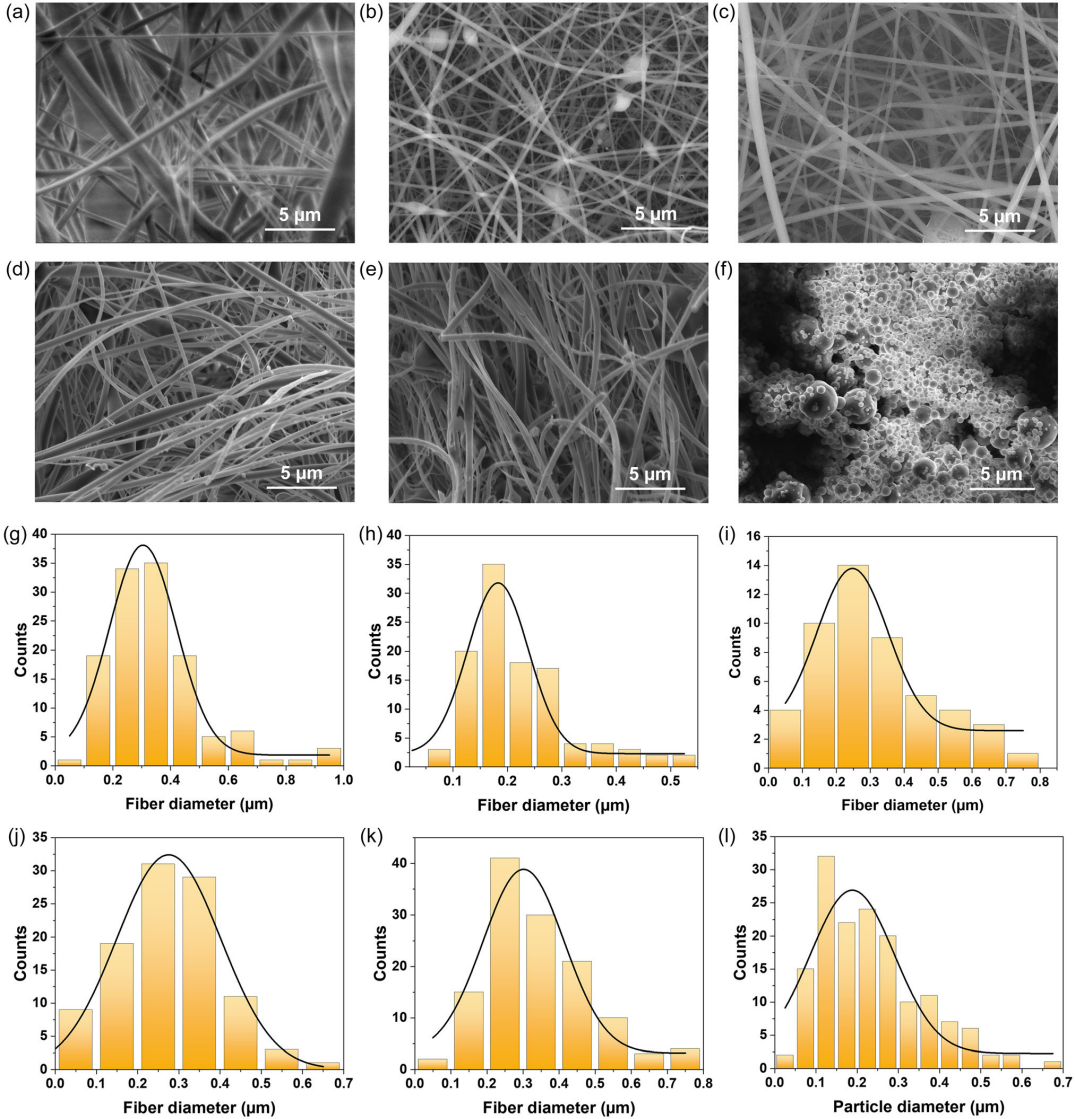
Scanning electron microscopic images and dimensional analysis of electrospun SKL–CA materials. Electrospinning of SKL–CA solutions with following composition in wt.% (a) 0:100, (b) 50:50, (c) 60:40, (d) 70:30, (e) 80:20, and (f) 100:0. Gaussian distribution of fiber/particle diameters based on scanning electron microscopic images of electrospun materials with the following compositions in wt.%. (g) 0:100, (h) 50:50, (i) 60:40, (j) 70:30, (k) 80:20, and (l) 100:0.

The effect of SKL–CA composition on fiber formation and morphology during electrospinning was systematically examined using scanning electron microscopy. Figure 2a–e displays the SEM images of the morphology of the electrospun/electrosprayed SKL–CA solutions with composition 0:100, 50:50, 60:40, 70:30, 80:20, and 100:0 wt.% respectively. SEM analysis shows uniform and continuous fibers for SKL contents between 0 and 80 wt.%, when electrospun with CA as a binder polymer, demonstrating stable fiber formation across a wide lignin composition range, as evident from Figure 2a–e. From the attempt to use SKL in the absence of CA, it is evident that electrospinning requires polymeric substances that can undergo molecular entanglements, since SKL alone only produced electrosprayed particles (Figure 2f). Similar observations have been previously published with hardwood kraft lignin [34]. In addition to its rheological properties that allow electrospinning, CA improves the processability and spinnability of the electrospinning solutions through molecular interactions such as H‐bonding with SKL [40]. Moreover, binders that participate in H‐bonding with lignin typically improve miscibility and spinnability of lignin solutions which also results in the formation of uniform continuous fibers [41, 42].

The diameter of electrospun nanofibers is a crucial parameter as it governs the surface area as well as the porosity of the resulting nanofiber mat, and it is influenced by several factors such as the distance between the spinneret and the collector plate, applied voltage, concentration of the spinning solution, flow rate, conductivity of the spinning solution, etc [43]. Gaussian distributions of the fiber/particle diameter are given in Figure 2g–l. The mean diameter of the fibers formed by CA alone was 304 nm, and for the SKL–CA compositions of 50:50, 60:40, 70:30, and 80:20 wt.%, the mean fiber diameters were 183, 248, 275, and 301 nm, respectively. For SKL electrospraying, the mean particle diameter was found to be 188 nm, with a homogenous particle size distribution, meaning electrospraying can be efficiently employed for the formation of lignin nanoparticles. Quantitative analysis of the fiber diameters revealed that all lignin‐containing fibers fall within the sub‐micrometer range, with only moderate variations observed as the SKL content increases. The similarity in fiber diameters across different SKL content suggests that the solution properties remained within a stable electrospinning regime, which enabled effective electrospinning jet stretching, despite the higher loading of SKL. The fiber diameter depends on electrospinning parameters as well as the type of the lignin and the concentration of the spinning solution [44, 45]. For example, hardwood kraft lignin from eucalyptus/CA and hardwood kraft lignin from poplar/CA systems exhibited comparable nanofiber diameters and porosity due to their similar solution characteristics, whereas hardwood kraft lignin from olive tree pruning (OTP)/CA produced significantly thinner fibers due to the higher shear viscosity and electrical conductivity of the spinning solutions [34].

**FIGURE 3:**
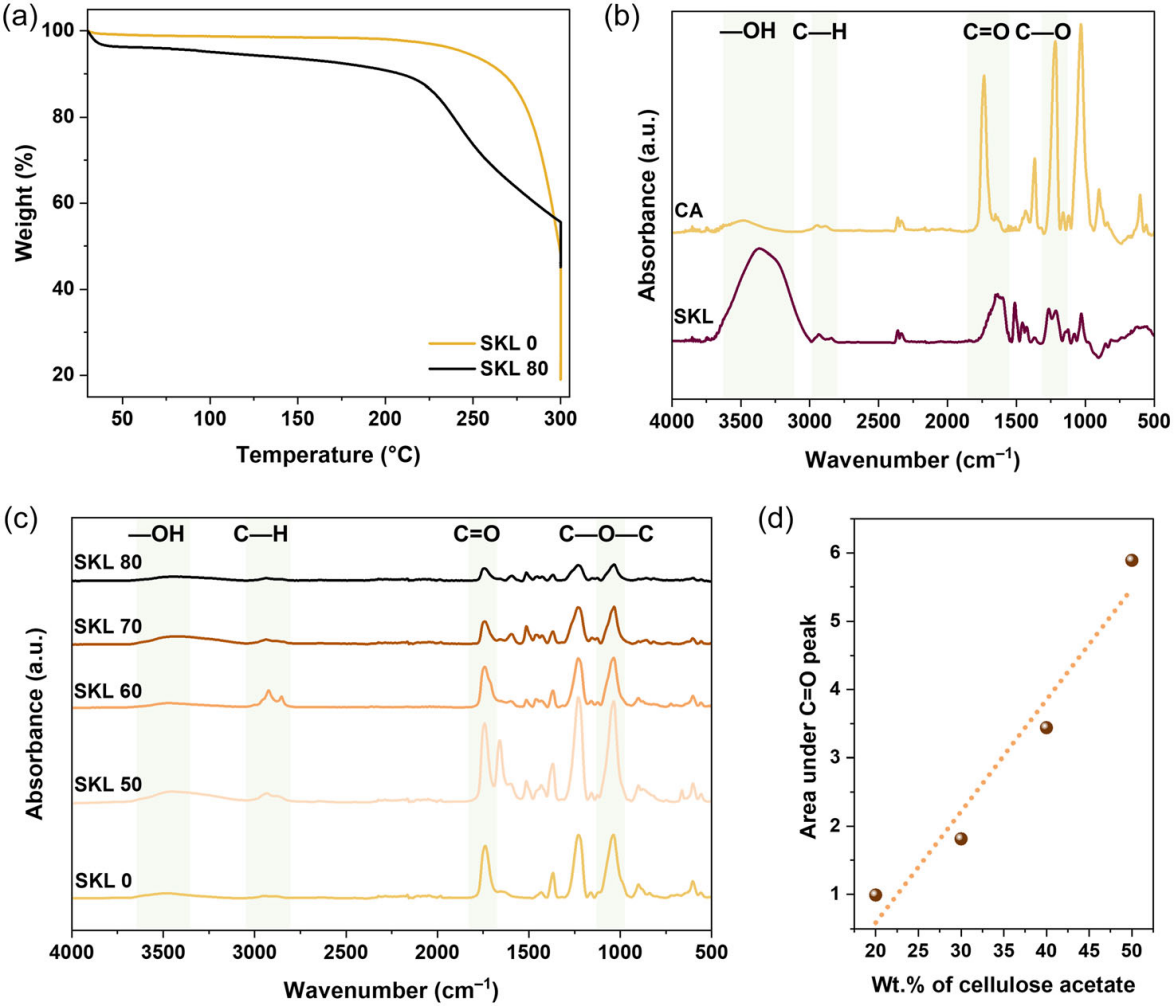
Thermal and spectroscopic properties of SKL–CA nanofiber mats with different compositions. (a) TGA thermogram in air. ATR‐FTIR spectra of (b) CA and SKL, and (c) electrospun products from SKL–CA at different composition. (d) Integrated band area at 1700–1790 cm^–1^ from (c) as a function of the theoretical cellulose acetate content.

The thermal degradation of the electrospun fibers was investigated by TGA to evaluate their thermostabilization behavior. Samples were heated in air from room temperature to 340°C at a heating rate of 2°C/min, followed by an isothermal hold at 340°C for 60 min. Pristine CA exhibited a single‐step degradation with an onset temperature of approximately 260°C, resulting in a total weight loss of 82.8% (Figure S1). In contrast, SKL displayed a two‐step degradation profile. The first weight‐loss event occurred around 70°C and corresponded to moisture loss, accounting for approximately 30% of mass loss. The second step started gradually at around 200°C, leading to an additional weight loss of approximately 17% by 340°C. During the subsequent isothermal hold, a further 13% mass loss was observed, resulting in a total weight loss of approximately 60% for SKL. Similarly, the electrospun fibers exhibited a multistep degradation behavior. An initial weight loss of 1%–5% was observed at around 60°C, which is attributed to the removal of adsorbed moisture. This was followed by a gradual weight loss starting at approximately 150°C, corresponding to the slow evaporation of residual solvent, contributing an additional ∼7% mass loss. A more pronounced decrease in weight was then observed between 250°C and 340°C, associated with the onset of thermal degradation. Notably, the overall weight loss decreased slightly with increasing SKL content in the fibers. Electrospun fibers composed solely of CA showed thermal degradation behavior like that of pristine CA (Figure 3a), with a total weight loss of approximately 80%. In contrast, fibers containing SKL exhibited significantly lower total weight loss (∼60%), indicating enhanced thermal stability upon incorporation of lignin (Figure S1).

ATR‐FTIR spectroscopy was used to identify and compare the characteristic functional groups of SKL, CA, and the electrospun fibers. For SKL, a wide band around 3000–3400 cm^–1^ was assigned to phenolic and aliphatic –OH stretching vibrations (Figure 3b). A band near 2937 cm^–1^ is attributed to C—H stretching in the methyl and methylene groups of the side chains and aromatic methoxy groups [46, 47]. The band around 1034 cm^–1^ is attributed to C—O deformation mainly in primary alcohol, aliphatic ethers, as well as C—H in aromatic rings. The band around 1215 cm^–1^ is assigned to C—C, C—O, and C═O stretching, mainly from condensed guaiacyl units. The narrow band at 1514 cm^–1^ is assigned to aromatic ring vibrations [48]. As shown in Figure 3b, the ATR‐FTIR spectra of CA have two distinct bands at around 1735 and 1215 cm^–1^ corresponding to stretching vibration modes of C═O of the acetyl group and C—O stretching vibrations, respectively [49]. The peak at 1026 cm^–1^ is assigned to the C—O—C asymmetric stretching of the ether group in the pyranose ring. The less intense band at 900 cm^–1^ is due to the C—H out of plane deformation [50]. ATR‐FTIR spectra of electrospun SKL–CA fibers are shown in Figure 3c. Compared to the individual components, similar but less intense bands were observed for the electrospun SKL–CA fiber mats. A decrease in intensity and broadening of the –OH stretching band is observed at around 3500 cm^–1^, suggesting possible H‐bonding between SKL and CA. Because of the interactions between –OH group of SKL and C═O groups of CA, the O—H bond weakens as the electron density of the –OH group is shared and which results in the decrease of vibrational frequency [51]. The weak band at ∼3000 cm^–1^ is related to the C—H stretching in methyl and methoxy groups of SKL and CA [52]. The intense band around 1750 cm^–1^ is attributed to the C═O stretching vibrations of acetyl groups from CA. Integration of the spectra within 1700–1790 cm^–1^ showed a linear correlation to the CA content in SKL–CA electrospun fibers (Figure 3d). The band around 1500 cm^–1^ corresponds to the aromatic skeletal vibrations from guaiacyl units of SKL, and 1232 cm^–1^ corresponds to the C—C, C—O, and C═O stretch mainly from condensed guaiacyl units. The band at 1040 cm^–1^ is attributed to the C—O deformation mainly from primary alcohol, aliphatic ethers, and C—H in aromatic rings, and the peak around 890 cm^–1^ corresponds to C—H deformation out of the plane of the hydrogens from p‐hydroxyphenyl units [48].

### 2.2 Adsorption of Methylene Blue by Electrospun Nanofibers

**FIGURE 4:**
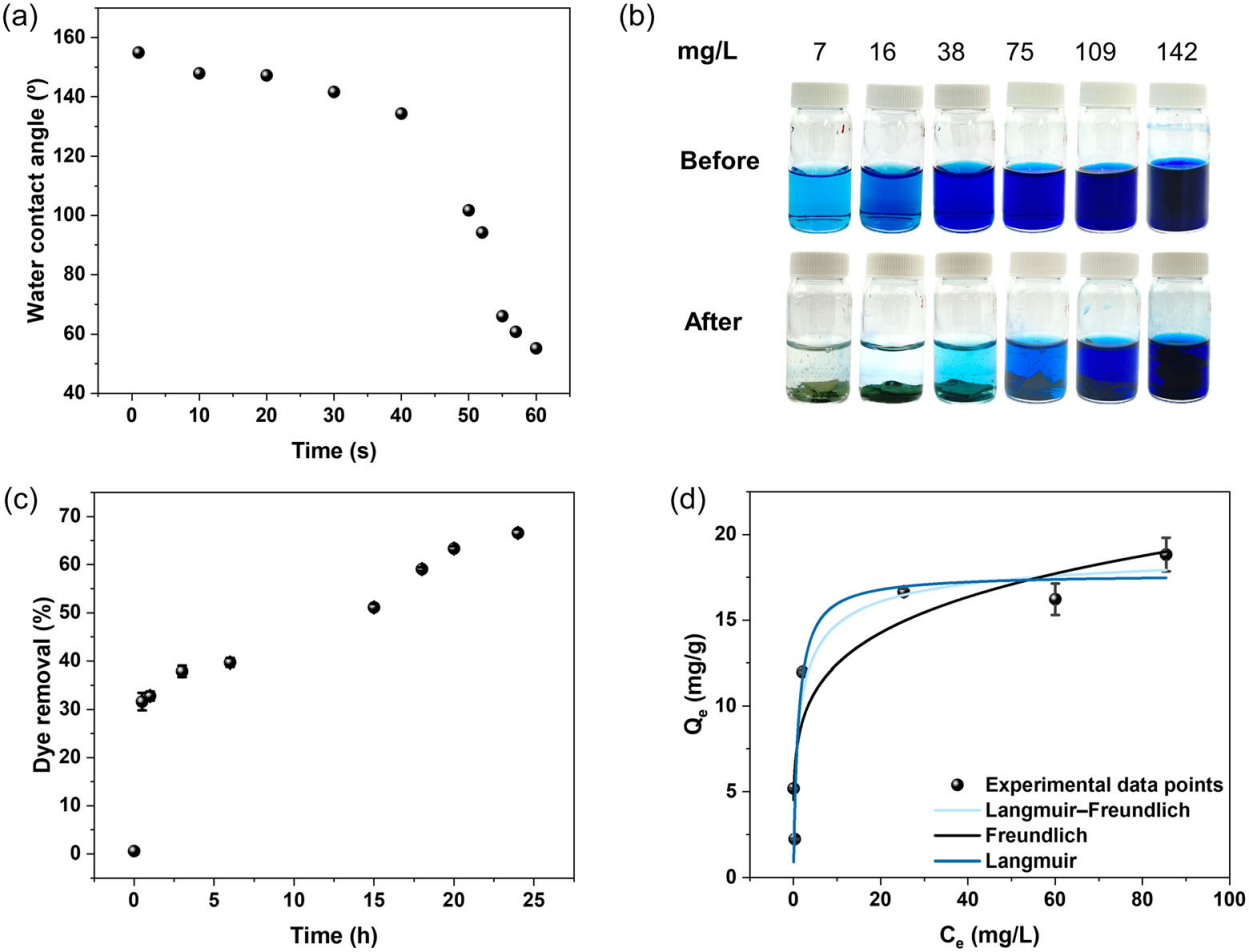
Dye adsorption application. (a) Water contact angle measurements of 80:20 electrospun fiber mat recorded as a function of time, (b) digital images showing methylene blue dye solutions of different initial concentrations before and after 24 h of adsorption by 80:20 electrospun fibers, (c) adsorption kinetics of methylene blue dye (*C*_0_ = 75 mg/L) on 80:20 electrospun fibers showing % dye removal as a function of contact time varying from 0–24 h, and (d) equilibrium adsorption isotherms showing equilibrium adsorption capacity (*Q*_e_, mg/g) as a function of equilibrium dye concentration (*C*_e_, mg/L) with Langmuir, Freundlich, and Langmuir–Freundlich model fits for dye adsorption after 24 h using initial methylene blue dye concentrations of 7–142 mg/L using 80:20 electrospun fiber mat.

The electrospun SKL–CA nanofiber mats with the highest lignin content (80 wt.%) were used as an adsorbent material for methylene blue, which is a cationic dye. Prior to the adsorption experiment, the water contact angle of the nanofiber mat was assessed. Although SKL and CA contain polar groups, such as hydroxyl functional groups, the high surface roughness of the nanofiber mats resulted in a static water contact angle of 155°, which reduced to 55° in 60 s (Figure 4a). Similar behavior has been reported for an electrospun lignin/ZnO nanofibrous membrane [53]. To facilitate the surface wetting for dye adsorption, electrospun nanofibers taken in the vial containing dye solutions were hand‐shaken a few times before placing them in the shaking incubator. Figure 4b shows the digital images of dye solutions before and after contact with electrospun nanofibers for 24 h. Adsorption kinetics were performed using an initial dye concentration of 75 mg/L for 24 h. The kinetics revealed a rapid dye uptake (∼32%) within the first 30 min, followed by a slower diffusion‐limited adsorption stage that continued over approximately 24 h, resulting in a comparable additional dye removal, as shown in Figure 4c. The adsorption kinetics curve suggests that readily accessible adsorption sites are occupied first, within the first 30 min, while subsequent adsorption is governed by slower mass transport processes. The absence of a sharp increase in dye removal over the period of the kinetics experiment indicates that strong electrostatic interactions are not dominant in this system, and the adsorption is mainly physically driven. This physical interaction can be weak interactions, such as van der Waals forces or π–π interactions between aromatic moieties of SKL and methylene blue dye molecules. This is further corroborated by the lower values for adsorption capacities that we found compared to the literature. For instance, when modified Indulin AT samples were used for methylene blue dye removal, with a maximum adsorption capacity of up to 55 mg/g, 61% of the interaction between the dye molecule and lignin was found to be H‐bonding and 38% through electrostatic interactions [54]. However, when lignin extracted from sugarcane bagasse was employed for methylene blue removal, the maximum adsorption capacity was determined to be 14 mg/g, because of the weak binding between the adsorbent and the adsorbate [55].

Lower dye concentrations of 7, 16, and 38 mg/L showed significant dye removal efficiency of 95.7, 99.6, and 94.5%, respectively with equilibrium adsorption capacities of 2.2, 5.2, and 12.0 mg/g, respectively. For higher concentrations such as 75, 109, and 142 mg/L, the dye removal efficiency was reduced to 66, 45, and 40%, respectively, with equilibrium adsorption capacities of 16.6, 16.2, and 18.8 mg/g, respectively (Figure 4d). A plateauing trend in adsorption capacity was observed for initial dye concentration ≥75 mg/L, suggesting the saturation of accessible adsorption sites. Equilibrium adsorption data were analyzed using Langmuir, Freundlich, and Langmuir–Freundlich adsorption isotherm models to gain insight into the adsorption mechanism, and the corresponding fitting results are summarized in Table S1. The Langmuir model assumes monolayer adsorption [56] and suggests that adsorption initially occurs at energetically favorable sites, as seen from Figure 4d. In contrast, the Freundlich model describes adsorption on a heterogenous surface with varying adsorption energies [55]. This surface heterogeneity is expected for the electrospun fiber mats as well as for SKL because of the diverse functional groups in lignin. The combined Langmuir and Freundlich model that fits the experimental data suggests that methylene blue adsorption on electrospun fiber mats involves monolayer adsorption at lower equilibrium concentrations followed by the slow occupation of heterogenous sites at higher equilibrium concentrations.

### 2.3 Influence of Thermostabilization on Fiber Fusion

**FIGURE 5:**
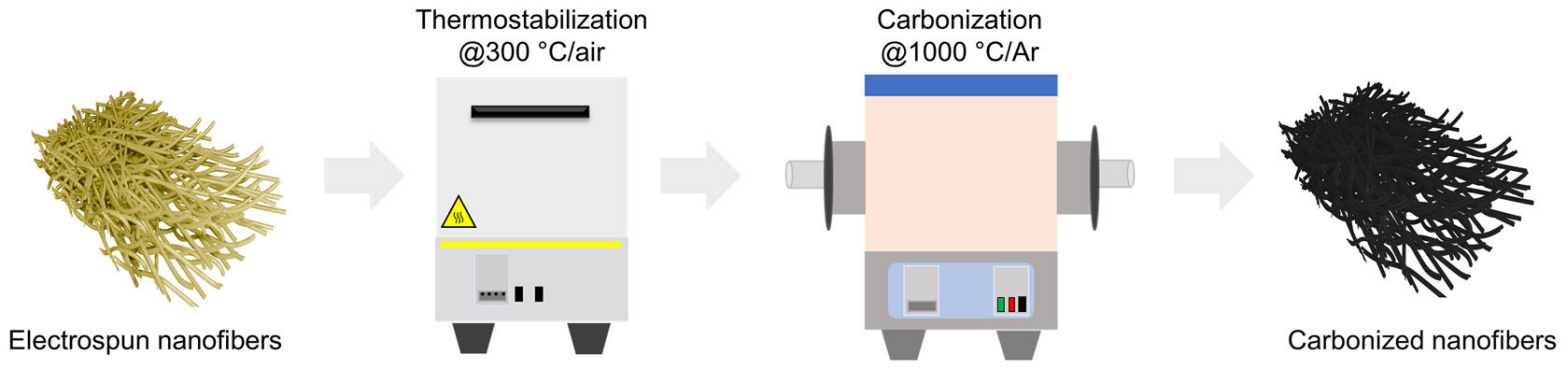
Illustration of the procedure used for thermostabilization and carbonization of electrospun SKL–CA nanofibers.

The goal of the thermostabilization method was to limit melting of lignin in SKL–CA electrospun nanofibers during carbonization, with the intention of enhancing the total pore volume. The procedure followed for thermostabilization and carbonization of the SKL–CA electrospun nanofibers with different lignin contents is presented in Figure 5.

Oxidative stabilization of nanofibers induces chemical changes such as dehydration, oxidation, and cross‐linking [57], which are essential for preserving fiber morphology during the subsequent carbonization step. It is also expected that residual solvent traces will leave the system during thermostabilization, which will eventually increase the porosity of the carbonized fibers. A stabilization temperature of 300°C was selected based on the TGA results (Figure 3a), where electrospun CA nanofibers begin to thermally degrade at ∼300°C and SKL–CA nanofibers begin to slowly degrade at ∼280°C. This temperature marks the optimum compromise temperature at which lignin undergoes effective oxidative crosslinking, improving thermal stability upon carbonization [58, 59]. The digital images of thermostabilized fibers as well as carbonized fibers corresponding to all the prepared compositions are showcased in the Figure S3. In the present study, nanofiber mats with higher lignin content (SKL:CA 70:30 and 80:20) exhibited effective thermostabilization, maintaining the majority of the fiber shape after stabilization at 300°C when the heating rate was 2°C/min (Figure S4 and S5). The thermostabilization yield (*Y*_TS_, %) of these samples was 66% and 64%, respectively, with respect to the initial weight of the fiber mats. In contrast, nanofiber mats with higher CA content (SKL:CA 0:100, 50:50, and 60:40) exhibited insufficient thermostabilization, characterized by significant fiber fusion during oxidative treatment (Figure S6 and S7). This behavior is attributed to the thermoplastic nature of CA, which softens under stabilization conditions [60]. The thermostabilization yields (*Y*_TS_, %) of these materials were 58% and 60%, respectively. For thermostabilized fibers from pure CA, *Y*_TS_ was found to be 33%, which is the lowest yield among all the thermostabilized fibers with different compositions of SKL therein. Importantly, the thermoplastic softening of CA during thermostabilization can be viewed as a sacrificial process that facilitates internal phase rearrangement, which contributes to the development of porosity upon subsequent carbonization, while lignin serves as the primary structure‐retaining component. Despite the loss of fibrous network, CA contributed substantially to the surface area more than SKL did. Similar observations have been reported in lignin–PLA electrospun systems, where the thermoplastic PLA component underwent phase separation during stabilization and subsequently acted as a sacrificial polymer, enhancing the porosity of carbon fibers formed [32].

### 2.4 Carbonized Electrospun SKL–CA Nanofibers

**FIGURE 6:**
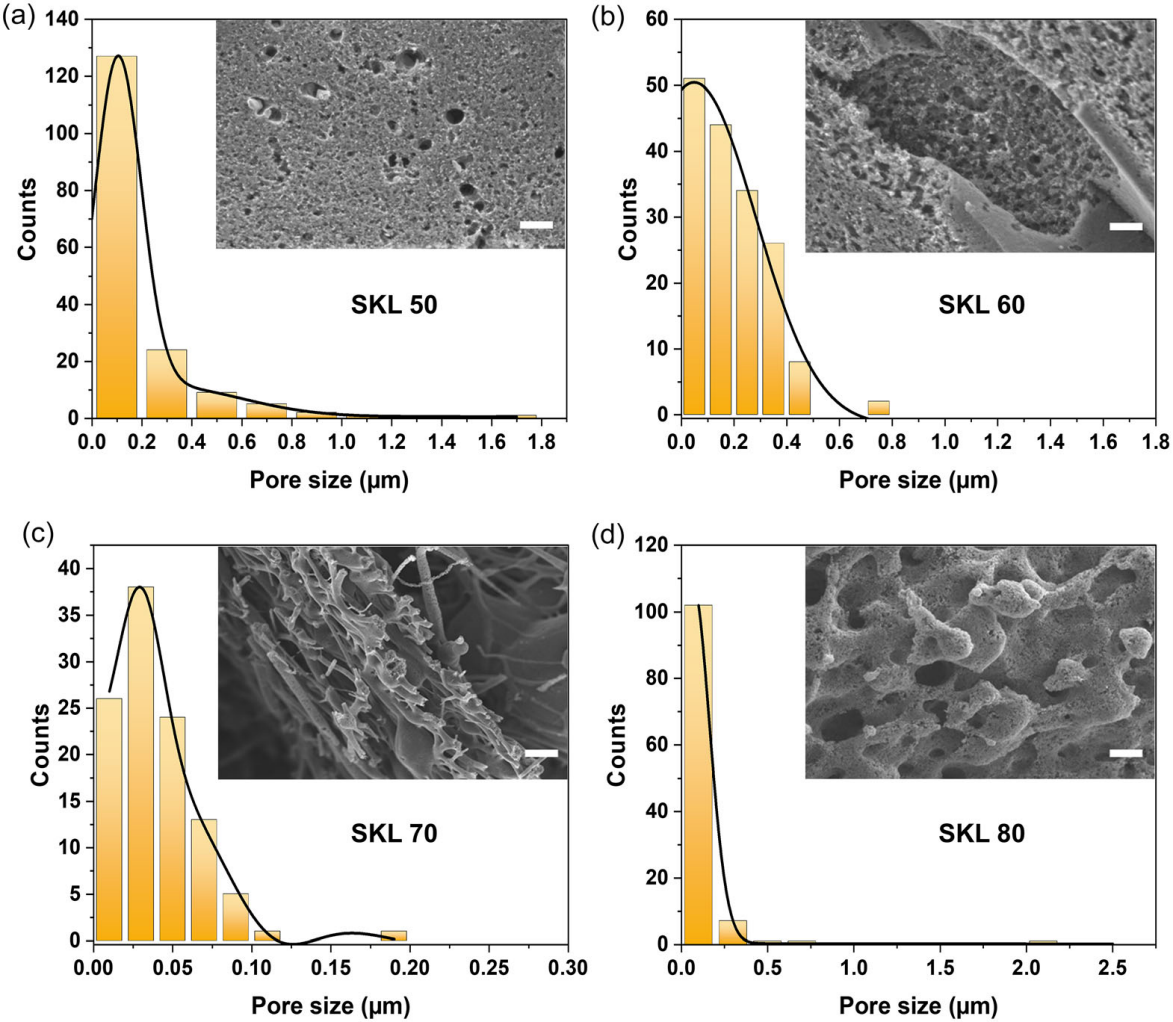
Pore size analysis of carbonized SKL–CA electrospun fibers. Pore size distributions with Gaussian fitting curves for carbonized SKL–CA nanofibers with composition in wt.% (a) 50:50, (b) 60:40, (c) 70:30, and (d) 80:20. Inset in each panel shows the corresponding SEM images of the fibers and the scale bars in the SEM images denote 2 µm.

Carbonization of the thermostabilized fibers was carried out in an inert atmosphere, by heating the sample up to 1000°C with a heating rate of 5°C/min, with an isothermal hold for 60 min at 1000°C, followed by cooling at 10°C/min to room temperature. The yield of carbonization with respect to the initial electrospun fiber mat weight, *Y*_C_ (%) is given in Table 1, which ranged from 0.8%–36%, generally increasing with increasing lignin content. The SEM images of the carbonized fibers, along with the Gaussian distribution of pore sizes are presented in Figure 6a–d, and the size of the pores was analyzed from the corresponding SEM images using ImageJ. All fibers exhibited a wide range of pore size distribution ranging from 0.01–0.1 µm. Carbonized fibers with higher lignin content maintained the fiber shape compared to carbonized fibers with lower lignin content or no lignin at all. Additional images of carbonized fibers with SKL–CA composition 50:50, 60:40, 70:30, and 80:20 wt.% are shown in Figure S8, S9, S10, and S11, respectively. A SEM image of the carbonized pure CA fiber is presented in Figure S7, revealing the high porosity of the carbonized material.

**TABLE 1:** Thermostabilization and carbonization data. Thermostabilization yield (*Y*_TS_), carbonization yield (*Y*_C_), carbonization yield with respect to thermostabilization (*Y*_C/TS_), BET surface area, micropore volume and atomic % of C, N, and O from EDS of carbonized SKL–CA nanofiber mats with different compositions in wt.%. The ± range of the atomic % of elements is obtained from the software SMILE VIEW used to analyze the EDS signal.

**SKL**–**CA** **Ratio, wt.%**	***Y***_**TS,**_ **%**	**Y**_**C,**_ **%**	**Y**_**C/TS,**_ **%**	**BET SA,** **m** ^**2**^ **/g**	**Micropore volume,** **cm** ^**3**^ **/g**	**C, at**–**%** a	**N, at**–**%** a	**O, at**–**%** a
0:100	33	0.8	2	—	—	—	—	—
50:50	58	9	15	773 ± 9	0.21	93.8 ± 0.1	2.74 ± 0.2	2.85 ± 0.0
60:40	60	17	29	536 ± 8	0.14	87.5 ± 0.9	0.5 ± 0.6	10.6 ± 0.8
70:30	66	36	55	63 ± 12	0.02	90.1 ± 0.3	4.3 ± 0.4	4.9 ± 0.2
80:20	64	35	55	41 ± 8	0.01	88.2 ± 0.2	4.9 ± 0.3	6.0 ± 0.1

a Atomic percentage from EDS.

To shed light on the porous carbons, their BET surface areas were determined by N_2_ adsorption. The measured BET surface area ranged from 41–773 m^2^/g and decreased with increasing lignin content, as it restricts the pore development observed from the SEM images and micropore volume analysis. A higher content of CA was found to be essential for the development of pores. Specifically, fibers containing 80, 70, 60, and 50 wt.% SKL exhibited BET surface areas of 41, 63, 536, and 773 m^2^/g, respectively (Table 1). The micropore volume of the porous carbons ranged from 0.01–0.21 cm^3^/g. The micropore volume increased with respect to the CA content in the precursor fibers, which acts as a sacrificial polymer, leaving high porosity. The trend of decreasing micropore volume with higher lignin content was in accordance with the BET surface area measured. Based on EDS mapping of carbonized SKL–CA electrospun fiber mats, their carbon content ranged from 88% to 94% (Table 1). EDS maps of carbonized fibers with SKL–CA composition 50:50, 60:40, 70:30 and 80:20 wt.% are given in the supplementary figures Figure S8, S9, S10 and S11, respectively.

**FIGURE 7:**
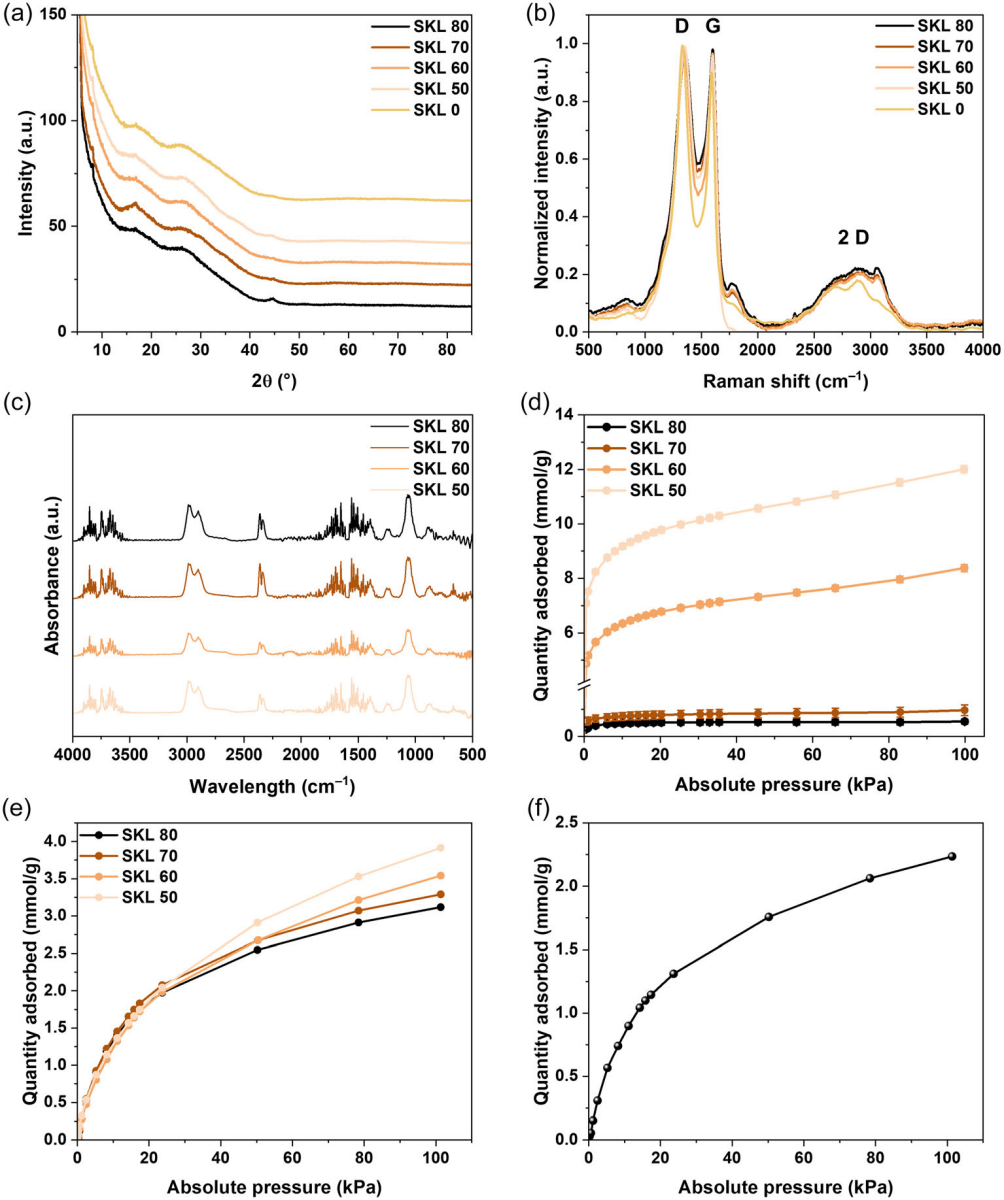
Structural characterization and nitrogen/carbon dioxide adsorption on carbonized SKL–CA nanofibers. (a) X‐ray diffractogram, (b) Raman spectra, (c) ATR‐FTIR spectra, (d) N_2_ adsorption, (e) CO_2_ adsorption isotherm at 0°C of the carbonized SKL–CA nanofibers with different composition in wt.%, and (f) CO_2_ adsorption isotherm at 0°C of the carbonized methylene blue adsorbed SKL–CA nanofibers with composition 80:20 in wt.%.

The structural evolution of SKL–CA fiber mats during thermostabilization and carbonization was analyzed using XRD, Raman spectroscopy, ATR‐FTIR, and solid‐state ^13^C MAS NMR. Figure S1 shows the X‐ray diffractogram of pristine CA and SKL. The XRD pattern of CA exhibits the usual peaks around *2θ* = 17° and 21° [61], which is typical for semicrystalline materials [62], and SKL exhibits a broad peak around *2θ* = 20° indicating its amorphous nature [63]. The XRD pattern of the thermostabilized fibers maintained the peaks at *2θ* = 17° and 21°, meaning the crystallinity of the fibers was not affected by the thermostabilization at 300°C. In the XRD pattern of the carbonized SKL–CA fibers (Figure 7a) a new peak around *2θ* = 44° was observed compared to the thermostabilized fibers, which is attributed to the reflection of the (100) plane of amorphous carbon [64].

Raman spectra of carbonized fibers are shown in Figure 7b. The Raman spectra show two clear and distinct intensity regions, ∼1330 cm^–1^, namely the D band, and ∼1590 cm^–1^, namely the G band. The broad peak between 2600–3000 cm^–1^ is also observed, corresponding to the 2D band. The D peak corresponds to the sp^2^ breathing modes of the aromatic rings, and the G peak corresponds to the in–plane bond stretching of sp^2^‐hybridized carbon atoms in both rings and chains [65, 66, 67], where the 2D band arises from a second‐order two‐phonon process [68]. Deconvoluted fits for all the Raman spectra collected are presented in the Figure S12. The intensity ratio of the D band to the G band gives semiquantitative information about the graphitic content. For this work, I_D_/I_G_ ratio for all the carbonized fiber mats prepared was close to unity, more precisely 1.1, 1.02, 0.99, 1.02, and 0.99, respectively, for carbonized fiber mats with SKL–CA composition 0:100, 50:50, 60:40, 70:30, and 80:20 wt.% respectively, which has previously been reported for lignin‐derived carbon fibers in the literature [22, 60].

ATR‐FTIR spectra of carbonized SKL–CA fibers presented in Figure 7c are found to have a low signal‐to‐noise ratio. A strong band around 1060 cm^–1^ and another band around 1250 cm^–1^ are attributed to the presence of C—O—C stretching. The band between 2900–2990 cm^–1^ is attributed to C—H stretching. The wavenumber region 1400–1750 cm^–1^ contains bands assigned to C—N stretching, C—C stretching, and C═O stretching [69], and the region around 3500 cm^–1^, associated with N—H stretching, exhibits significant spectral noise.

### 2.5 Gas Adsorption Performance (CO_2_/N_2_ Adsorption Isotherm)

N_2_ adsorption isotherms were measured at 77.34 K and CO_2_ isotherms were measured at 273 K and 1.0 bar, which are presented in Figure 7d and e, respectively. At 273 K and 101 kPa, carbonized fibers from SKL–CA 50:50 wt.% exhibited CO_2_ uptake of 3.9 mmol/g and N_2_ uptake of 12.0 mmol/g. With higher lignin content in the precursor fibers, the CO_2_ uptake gradually decreased. Carbonized fibers with 60:40 wt.% exhibited CO_2_ uptake of 3.5 mmol/g and N_2_ uptake of 8.0 mmol/g. Carbonized fibers with 70:30 wt.% had a CO_2_ uptake of 3.3 mmol/g and N_2_ uptake of 1.0 mmol/g, and 80:20 wt.% showed a CO_2_ uptake of 3.1 mmol/g and N_2_ uptake of 0.5 mmol/g. N_2_ uptake for all the carbonized fibers was in accordance with the BET surface area measured, as reported in Table 1, but the CO_2_ uptake for all the carbonized fibers ranged from 3.1–3.9 mmol/g with decreasing SKL content. Interestingly, carbonized fibers with higher lignin content and low surface area showed equally good CO_2_ uptake as the carbonized fibers with lower lignin content and higher surface area. The observed correlation between BET surface area and N_2_ uptake was not similar for CO_2_ uptake, as the CO_2_ adsorption process is governed not only by the surface area but also by the surface chemistry and micropore volume. The carbon materials in the present study exhibited high CO_2_ uptake values, even though the carbonized fibers were not subjected to any further activation after fabrication [21, 70], or doping before/during [19, 71, 72] the carbonization process. Benchmarking of lignin‐derived carbon materials based on CO_2_ uptake values are given in Table S2. Across the literature, the highest CO_2_ uptake reported for lignin‐derived carbon is 13.6 mmol/g (298 K, 1–10 atm), which is achieved by KOH activation along with N‐doping using urea, which resulted in a BET surface area of 3000 m^2^/g. Moreover, previously reported lignin‐based carbon materials with activation demonstrated lower CO_2_ uptake values compared to those achieved in the present work [73, 74]. However, lignin‐based carbon materials without heteroatom doping or activation have always demonstrated lower CO_2_ uptake values [70, 75]. In the only study explicitly reporting CO_2_ sorption by carbonized lignin‐based electrospun fibers, a CO_2_ uptake of 1.56 mmol/g was reported; however, the fibers relied on a fossil‐derived binder polymer, poly(acrylonitrile), rather than a bio‐based alternative [76].

The shape of the CO_2_ adsorption isotherm provides mechanistic insight into the nature of gas–solid interactions and the chemical transformations occurring during thermostabilization and subsequent carbonization. The CO_2_ adsorption isotherm shown in Figure 7e indicates strong adsorbate–adsorbent interactions, evidenced by a steep initial CO_2_ uptake at low relative pressures and a clear deviation from a purely physisorptive adsorption profile, suggesting the contribution of chemisorptive interactions [77]. As native SKL contains negligible nitrogen, the observed effect may arise from nitrogen incorporation during thermostabilization, as DMF begins to decompose at elevated temperatures. Although DMF evaporates at ∼150°C and thermally degrades at ∼300°C, partial retention of nitrogen in the material cannot be entirely ruled out. This has been seen by the TGA analysis of thermostabilized fibers as shown in the Figure S1. The gradual decrease in weight percentage from 300°C is also attributed to the hydrolysis of DMF to amine functionalities [78] along with the degradation of lignin's backbone. At temperatures above 300°C, the primary decomposition of lignin occurs by homolytic cleavage of the C—C and C—O bonds, resulting in the formation of low‐molecular weight phenolic compounds such as guaiacol, methyl guaiacol, and ethyl guaiacol [79]. Around 400°C, homolysis of β–O–4 bonds produces phenoxy radicals [80]. The flattening of the thermogram in Figure S1 after 600°C can be attributed to aromatic condensation [79] The resulting amine functionalities from DMF decomposition after 300°C, even at lower percentages, may act as basic sites capable of enhancing CO_2_ affinity for the carbonized fibers, which has ∼0.5–5 atomic % of nitrogen as per the elemental analysis by EDS (Table 1, Figure S8, S9, S10, and S11), contributing to the observed CO_2_ sorption behavior.

The samples with higher lignin content (70 and 80 wt.%, with lower BET surface area) were found to have higher nitrogen content from the elemental analysis. Nitrogen functionalities significantly enhance the CO_2_ uptake. As a result, even with lower surface area and microporosity, the enhanced interaction between CO_2_ molecules and the N‐containing functionalities compensates for the reduced number of adsorption sites, which eventually leads to comparable overall CO_2_ uptake observed for the samples. It has been reported that lignin‐based nanofibers stabilized at 350°C exhibit a nitrogen content of 0.48%, whereas samples stabilized below 300°C show no detectable nitrogen. This was attributed either to the presence of residual DMF or to the protein residues inherently present in lignin [59]. Similarly, the solid‐state ^13^C MAS spectrum of carbonized SKL–CA nanofibers with 80 wt.% of SKL is shown in Figure S13, which has high spectral noise due to the highly conductive nature of the sample, and the peak at 125 ppm is typical for aromatic carbon atoms [81]. Due to the low signal‐to‐noise ratio, trace levels of nitrogen functionalities cannot be excluded solely based on the solid‐state ^13^C MAS spectrum, as EDS analysis revealed traces of nitrogen in the samples. Similar observations were made when zirconium–ellagate MOF was made using DMF as cosolvent, where DMF hydrolyzed to dimethylammonium cations, which was found in the pores of the MOFs after activation [78].

To investigate the potential for sequential material utilization, methylene blue‐loaded electrospun nanofibers were subjected to thermostabilization and carbonization to evaluate their potential repurposing as CO_2_ sorbents. Methylene blue adsorbed electrospun nanofibers with SKL content 80 wt.% were also subjected to thermostabilization followed by carbonization as per the protocol mentioned in Figure 5 to evaluate if the spent fibers can be repurposed as CO_2_ sorbents. The resulting carbonized fibers exhibited comparable CO_2_ sorption capacity (2.2 mmol/g) to that of carbonized fibers without dye uptake (3.1 mmol/g), as shown in Figure 7f. A slightly lower value of CO_2_ sorption capacity is observed for the carbonized spent fibers, due to the partial coverage or blockage of surface sites by residual species originating from the adsorbed dye during carbonization, which is further clarified from the SEM image of the carbonized spent fibers shown in Figure S2. From the SEM image of the carbonized spent fibers, it is observed that the surface is covered with residues originating from the methylene blue dye, and from the EDS mapping, it is observed that the surface has the presence of elements such as N, S, Cl, O, and C, where N, S, and Cl originate solely from the dye residues. The digital images of spent fibers before and after carbonization and the SEM image of spent fibers after thermostabilization are also shown in Figure S2.

## 3 Conclusions

This study demonstrates the incorporation of up to 80% of SKL in electrospun nanofibers without any chemical modification or solvent fractionation of the lignin. Electrospun nanofiber mats produced from mixtures of CA and SKL exhibited effective dye adsorption from aqueous solutions, with cationic methylene blue used as the model pollutant. The nanofibers with different compositions were subjected to carbonization at 1000°C in an inert atmosphere after thermally stabilizing them at 300°C in air. The variation in lignin content influenced both carbon yield and surface area, with higher lignin content yielding more carbon yield but lower surface area due to reduced CA content and restricted pore development. The CO_2_ adsorption behavior reflected a trade‐off between these parameters rather than a monotonic dependence on surface area. As an attempt to demonstrate multipurpose sequential use of the electrospun fiber mats, they were first used for methylene blue adsorption and subsequently carbonized for CO_2_ sorption with promising results. Future work will focus on understanding how CO_2_ sorption depends on the chemical structure of carbon materials derived from lignin‐based precursor nanofibers.

## 4 Experimental Section

### 4.1 Materials

SKL BioPiva100 was obtained from UPM (Finland), CA (degree of deacetylation 37%), acetone, and N, N′‐dimethylformamide (DMF) were purchased from Honeywell. Methylene blue was purchased from Sigma–Aldrich. All the chemicals were used as received.

### 4.2 Methods

#### 4.2.1 Electrospinning

SKL and CA were dissolved in a 1:2.4 v/v DMF/acetone mixture, at 20 wt. % total concentration, in different wt.% ratios; 0:100, 50:50, 60:40, 70:30, and 80:20 of SKL and CA, respectively. All solutions were prepared by magnetic stirring at 1000 rpm at room temperature for 24–36 h. Sonication was done if it was hard to dissolve the solids.

A volume of 20 mL was placed in a syringe fitted with a Hamilton point‐style 2 needle having a gauge size 21 and length 51 mm, which was attached to the holder in a horizontal configuration and coupled to a high‐voltage power supply providing 20 kV. The needle tip was placed 15 cm from an aluminum foil wrapped around a rotating drum collector, and a positively charged jet from the syringe tip was projected onto the negatively charged collector. Nanofibers were then carefully removed from the collector plate. All experiments were performed at room temperature. The polymer solution was dispensed at 0.04 mL/min. The electrospinning operation was carried out for 5–8 h to have enough material after carbonization.

#### 4.2.2 Dye Adsorption

Initially, dye adsorption experiments were carried out using methylene blue dye solutions with initial concentrations ranging from 7–142 mg/L. The dye solutions were made in deionized water. 30 mg of SKL–CA electrospun nanofibers with 80:20 composition (in wt.%) were placed in 10 mL of methylene blue dye solutions with different concentrations. Equilibrium concentrations of the dye solutions after dye adsorption were measured after 24 h of agitation at room temperature at a rate of 100 rpm (Incu‐ShakerTM, Fisherbrand). After adsorption, the fibers were separated via centrifugation (10000 rpm for 10 min using a Universal 320 centrifuge) and the supernatant was used for UV–vis absorbance measurements. Similarly, the kinetics of dye adsorption were determined by performing experiments using the above‐mentioned method by keeping the samples at different time intervals ranging from 0–24 h. The spent fibers were dried and used for thermostabilization followed by carbonization.

The equilibrium adsorption capacity (*Q*_e, adsorption_) was measured using the following equation [47]

(1) $$Q_{e,\text{adsorption}}=\left(C_{0\;}-\;C_{e\;}\right)\times \frac{V}{m}$$

Dye removal efficiency was calculated using the following equation

(2) $$\%\;Dye\;\text{Removal}\;=\frac{\left[C_{0\;}-C_{e\;}\right]}{C_{0}}\;\times 100$$

*C*_*o*_ is the concentration of the initial dye solution, *C*_*e*_ is the equilibrium concentration of the dye, *V* is the volume of the dye solution in L, and *m* is the mass of the adsorbent in g.

The experimental equilibrium data were fitted by applying Langmuir, Freundlich, and Langmuir–Freundlich models as per the Equations (3), (4) and (5), respectively [54].

(3) $$Q_{e}=\;\frac{Q_{max}\;\times \;K_{L\;}\times \;C_{e}}{1+\;K_{L}\times \;C_{e}}$$

(4) $$Q_{e}=K_{F\;}\times \;{C_{e}}^{1/n_{F}}$$

(5) $$Q_{e}=\;\frac{Q_{max}\;\times \;{\left(K_{LF\;}\times \;C_{e}\right)}^{n_{LF}}}{1+\;{\left(K_{LF\;}\times \;C_{e}\right)}^{n_{LF}}}$$

#### 4.2.3 Thermostabilization

The stabilization of SKL–CA electrospun nanofibers with different compositions (in wt.%) was performed using a muffle furnace (Nabertherm) with a heating rate of 2°C/min, to a final temperature of 300°C in air, fllowed by holding the sample isothermally for 1 h.

Yield after thermostabilization (*Y*_TS_, %) with respect to the initial weight of electrospun nanofibers was calculated using the following equation

(6) $$\text{Thermostabilization}\;\text{Yield}\;\left(Y_{TS},\;\%\right)\;=\frac{W_{1\;}}{W_{0}}\;\times 100$$

where *W*_0_ is the initial weight of electrospun nanofibers and W_1_ is the weight after thermostabilization.

#### 4.2.4 Carbonization

Carbonization of stabilized electrospun SKL–CA nanofibers with different compositions (in wt.%) was carried out in a tube furnace (Carbolite) under argon gas flow, by heating from 25°C–1000°C at 5°C/min, followed by a hold at 1000°C for 1 h.

Yield after carbonization (*Y*_C_, %) with respect to the initial weight of electrospun nanofibers was calculated using the following equation

(7) $$\text{Carbonization}\;\text{Yield}\;\left(Y_{C},\;\%\right)\;=\frac{W_{2\;}}{W_{0}}\;\times 100$$

where *W*_2_ is the weight after carbonization.

Similarly, yield after carbonization (*Y*_C/TS_, %) with respect to the weight of thermostabilized electrospun nanofibers was calculated using the following equation

(8) $$\text{Carbonization}\;\text{Yield}\;\left(Y_{C/TS},\;\%\right)\;=\frac{W_{2\;}}{W_{1}}\;\times 100$$

### 4.3 Characterization

#### 4.3.1 ATR‐FTIR Spectroscopy

Attenuated total reflectance‐Fourier transform infrared (ATR‐FTIR) spectroscopy was used for the analysis of functional groups in the SKL, CA, and electrospun and carbonized nanofiber samples with different compositions (in wt.%) of SKL–CA, by using a Varian 610‐IR Spectrometer equipped with diamond ATR Optics. The spectra were measured from 400–4000 cm^−1^ with a total of 32 scans.

#### 4.3.2 UV–Vis Spectrophotometry

Absorbance measurements of methylene blue dye solutions before and after adsorption using SKL–CA electrospun nanofiber 80–20 composition (in wt.%) were performed using a UV–Vis spectrophotometer (Genesys 150 UV–Vis spectrophotometer, Thermo Scientific) with cuvettes (path length *l* = 1 cm) at time 0 and after 24 h for obtaining the adsorption isotherm and at regular intervals for studying the adsorption kinetics, which was conducted at a wavelength *λ *= 664 nm.

#### 4.3.3 Water Contact Angle

A small piece of SKL–CA nanofibers with 80–20 composition (in wt.%) was fixed to a microscope slide for contact angle measurements. 2 μL of water was added to the surface using a Drop Shape Analyzer DSA25E (KRÜSS instruments, Germany). Measurements were recorded over time and analyzed using ADVANCE software (KRÜSS instruments, Germany).

#### 4.3.4 Thermogravimetric Analysis (TGA)

Thermogravimetric analysis (TGA) was used to study the thermal degradation behavior of SKL, CA, and electrospun nanofiber samples with different compositions (in wt.%) of SKL–CA using a Perkin Elmer TGA4 model with an aluminum oxide pan, using 5–20 mg of samples. The temperature ramp was from RT–300°C with a heating rate of 2°C/min in air and an isothermal hold at 300°C for 60 min. For thermostabilized samples, carbonization was also performed in the same instrument by heating the sample up to 1000°C in an N_2_ atmosphere with a heating rate of 5°C/min and an isothermal hold for 60 min at 1000°C.

#### 4.3.5 Raman Spectroscopy

The graphitic structure of carbonized electrospun nanofiber samples with different compositions (in wt.%) of SKL–CA was investigated using Raman spectroscopy, recorded on a Horiba Labram S3000 instrument via a 10x magnification lens using a 532 nm laser, with an acquisition time of 3 s and 25 accumulations in the range of 200–3999 cm^–1^.

#### 4.3.6 Imaging and EDS Analysis

A JSM‐IT 800 scanning electron microscope was used to image SKL–CA electrospun nanofibers with different compositions (in wt.%) and thermostabilized and carbonized samples. Electrospun samples and thermostabilized samples were coated for 60 s with gold using a JFC‐1200 fine coater before the SEM imaging. The gold particles added by sputtering are about 5–15 nm in size. The same instrument was used for elemental analysis of the carbonized samples in HV vacuum mode with an accelerating voltage of 15 kV, probe current ranging from 0.16 to 0.17 nA, and the working distance was maintained at 10–11 mm. The dead‐time was set to 3%. EDS signal was analyzed using SMILE VIEW software from JEOL. Digital photographs of the Electrospinning solutions, SKL–CA electrospun nanofibers with different compositions (in wt.%) and thermostabilized and carbonized samples were acquired using an iPhone 13 Pro digital camera.

#### 4.3.7 X‐Ray Diffraction (XRD)

XRD patterns of the SKL, CA, electrospun nanofiber samples with different compositions (in wt.%) of SKL–CA samples, and the thermostabilized and carbonized nanofiber samples were obtained using a Bruker D8 Discover Diffractometer in reflection mode using Cu Kα radiation (*λ *= 1.5418 Å), *2θ* ranging from 5° to 85° with an increment of 0.01 and a rotation speed of 15 rotations/min.

#### 4.3.8 CO_2_ Uptake and BET Surface Area

Micromeritics Gemini VII Series (model 2390a) was used to measure the Brunauer–Emmett–Teller (BET) surface area and CO_2_ adsorption capacity of the carbonized electrospun SKL–CA fibers. All samples were degassed at 150°C for 12 h. BET surface area measurement was performed using 90–190 mg of samples with N_2_ at 77.34 K using 20 points with relative pressure (p/p°) ranging from 0.005 to 0.98. Free space was evaluated using He gas, which is not adsorbed by the samples. The same samples were used for studying CO_2_ adsorption capacity after degassing at 150°C for 12 h. The temperature was kept constant at 0°C using an ice bath, and 18 points were measured with absolute pressure (kPa) ranging from 0.12–101 kPa.

#### 4.3.9 Solid‐State Nuclear Magnetic Resonance Spectroscopy (ss‐NMR)

Solid‐state ^13^C MAS spectrum of carbonized SKL–CA nanofibers with 80–20 composition (in wt.%) was collected at a magnetic field of 9.4 T with a Bruker Avance‐III spectrometer using a 4 mm probe head and a 12 kHz MAS rate. Acquisition involved a 90° excitation pulse of 3.2 µs (∼78 kHz rf nutation power) and 16 384 signal transients were collected with a relaxation delay of 5 s. No proton decoupling or cross‐polarization magic‐angle‐spinning (CPMAS) could be employed due to the highly conductive character of the sample, which hindered tuning/matching of the proton channel. ^13^C chemical shift is reported with respect to tetramethylsilane (TMS).

## Supporting Information

Additional supporting information can be found online in the Supporting Information section.

## Author Contributions

**Unnimaya Thalakkale Veettil**: conceptualization, methodology, investigation, validation, formal analysis, visualization, writing – original draft, writing – review and editing. **Fengyang Wang**: investigation, writing – review and editing. **Mirva Eriksson**: investigation, validation, writing – review and editing. **Aleksander Jaworski**: investigation, writing – review and editing. **Mika H. Sipponen**: funding acquisition, project administration, supervision, writing – review and editing.

## Funding

This study was supported by Vetenskapsrådet (2020−03752), H2020 European Research Council (101075487).

## Conflicts of Interest

The authors declare no conflicts of interest.

## Supporting information

Supplementary Material

## Data Availability

The data that support the findings of this study are openly available in Zenodo at https://doi.org/10.5281/zenodo.18753055.
