# Supplementary material for: Electrospinning of Softwood Kraft Lignin With Cellulose Acetate: Dye Adsorption, Carbonization, and Carbon Dioxide Capture
Source: ChemSusChem. 2026 May 15;19(10):e70717. doi: 10.1002/cssc.70717 (PMC13177172; doi:10.1002/cssc.70717)
Supplement: Supplementary file 1 — Supplementary Material [file CSSC-19-e70717-s001.pdf]

# Supporting Information

# Electrospinning of softwood kraft lignin with cellulose acetate: dye adsorption, carbonization and carbon dioxide capture

*Unnimaya Thalakkale Veettil* [a], *Fengyang Wang* [a], *Mirva Eriksson* [a], *Aleksander Jaworski* [a], *Mika H. Sipponen* [a] *

[a] *Department of Chemistry, Stockholm University, Svante Arrhenius väg 16C, 106 91 Stockholm, Sweden*

* *Corresponding author:* mika.sipponen@su.se

This PDF contains:

Table S1–S2

Supplementary Figures S1–S13

**Table S1.** Fitting equilibrium model parameters for methylene blue adsorption on electrospun fiber mat.

| **Model** | **$Q_m$ mg $g^{-1}$** | **$K_L$ $Lmg^{-1}$** | **$K_F$ $L^{1/nF}$ $mg^{1-1/nF}$ $g^{-1}$** | **$1/n_F$** | **$K_{LF}$ $Lmg^{-1}$** | **$n_{LF}$** | **$R^2_{adj}$** |
|---|---|---|---|---|---|---|---|
| Langmuir | 17.69 ± 1.5 | 0.92 ± 0.5 | | | | | 0.87 |
| Freundlich | | | 7.89 ± 1.6 | 0.19 ± 0.05 | | | 0.84 |
| Langmuir–Freundlich | 19.50 ± 4.9 | | | | 0.65 ± 0.91 | 0.61 ± 0.38 | 0.87 |

**Table S2.** Benchmark comparison of $CO_2$ uptake performance of carbonized SKL–CA nanofibers developed in this work with previously reported lignin-derived carbon materials.

| **Type of lignin** | **Technique** | **Pre/post-treatment** | **BET surface area ($m^2$/g)** | **$CO_2$ adsorption capacity (mmol/g)** | **Reference** |
|---|---|---|---|---|---|
| Alkali lignin (Solar bio, Beijing) | Pyrolysis @750 °C, $N_2$ | Post treatment: Acid (10% HCl) washing assisted with ultrasound | 978─1134 | 3.5─4.0 (25 °C) | [1] |
| Alkali lignin (Solar bio, Beijing) | Pyrolysis @750 °C, $N_2$ | N/A | 118 | 0.68 (25 °C) | [1] |
| Alkali lignin (Solar bio, Beijing) | Negative pressure Pyrolysis @800 °C, $N_2$ | Post treatment: Acid (5% HCl) washing assisted with ultrasound | 1577 | 3.6 (25 °C) | [2] |
| Alkali lignin (Solar bio, Beijing) | Negative pressure Pyrolysis @800 °C, $N_2$ | N/A | 216 | 0.28 (0 °C) | [2] |
| Corn straw lignin | Pyrolysis @300─600 °C, $N_2$ | $H_3PO_4$ activation | 820 | 0.4 (0 °C) | [3] |
| Enzymatic hydrolysis lignin | Microwave @800 MW | Pretreatment: Humified $N_2$, mixed with KOH | 480─2870 | 0.57─1.31 (30 °) | [4] |
| Softwood kraft lignin (BioPiva 100, Indulin AT) | Pyrolysis @600─900 °C, $N_2$ | Oxidative thermostabilization of particles in the presence of 2.5 wt% of CNF and physical activation using $NH_3$ steam | 1152 | 1.75 (40 °C) | [5] |
| Dealkaline lignin (TCI America) | Pyrolysis @800 °C, $N_2$ | Pretreatment: Grinded with $Na_2S_2O_3$ and KOH | 741─3626 | 0.75─10 (25°C) | [6] |
| Enzymatic hydrolysis lignin | Mechanochemical treatment and low-temperature activation @600 °C | Pretreatment: Mixed with Melamine/Ultrasonication/Hydrothermal treatment; Post treatment: Washed with HCl | 602─2030 | 3.26─5.0 (0 °C) | [7] |
| Softwood kraft lignin (UPM BioPiva 100) | Pyrolysis @800 °C, $N_2$ | Pretreatment: Mixed with urea and $HNO_3$ | 1000 | 1.4 (20 °C) | [8] |
| Softwood kraft lignin (UPM BioPiva 100) | Pyrolysis @1000 °C, $N_2$ | Electrospinning with cellulose acetate, Oxidative thermostabilization | 33─781 | 3.1─3.9 (0 °C) | This work |

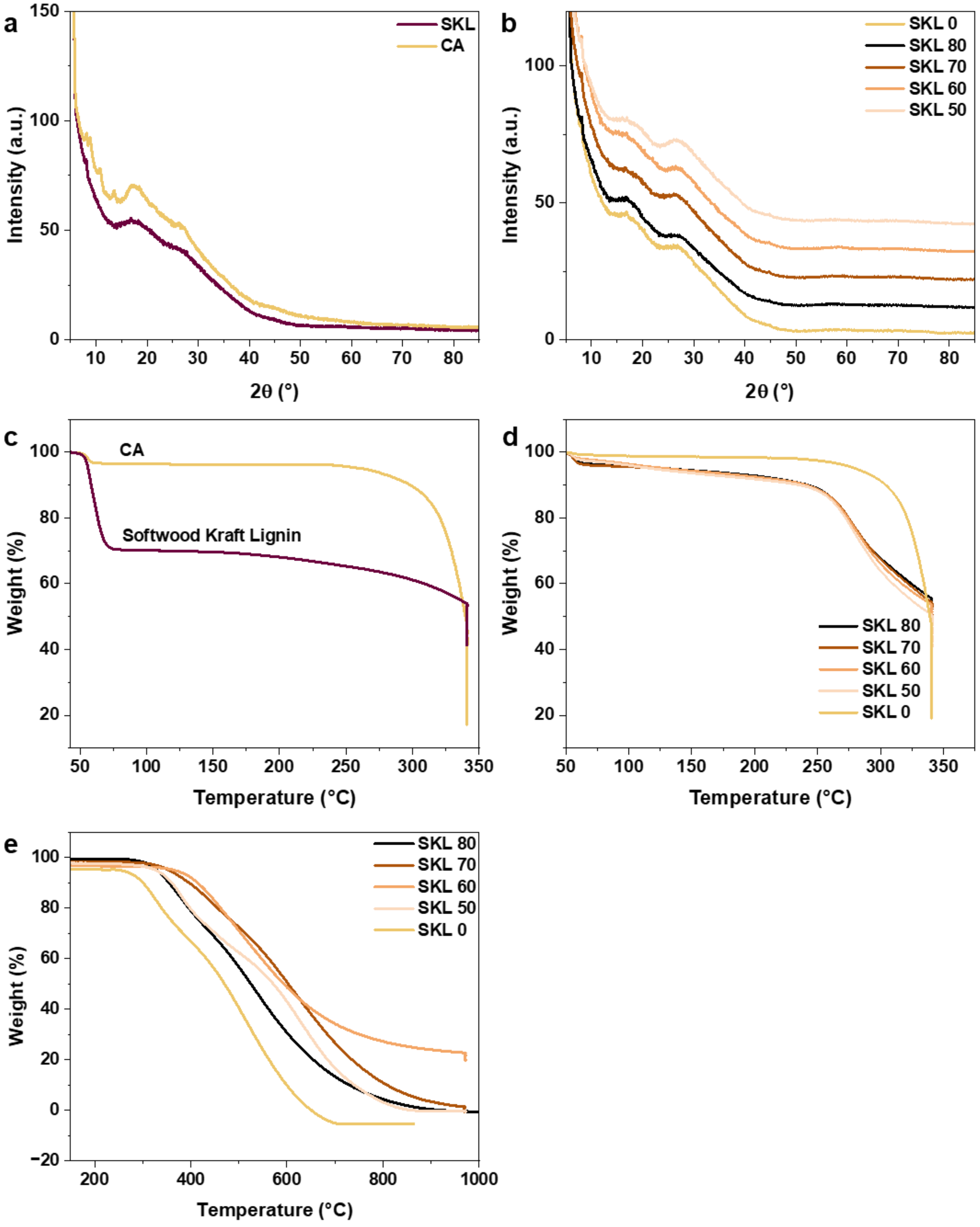


**Figure S1.** X-Ray diffractogram (a) pristine CA and SKL and (b) thermostabilized SKL–CA nanofibers with different composition in wt.%, Thermogram of (c) pristine CA and SKL in air, (d) SKL–CA nanofibers with different composition in wt.% in air and (e) Thermogram of thermostabilized SKL–CA nanofibers with different composition in wt.% in inert atmosphere.

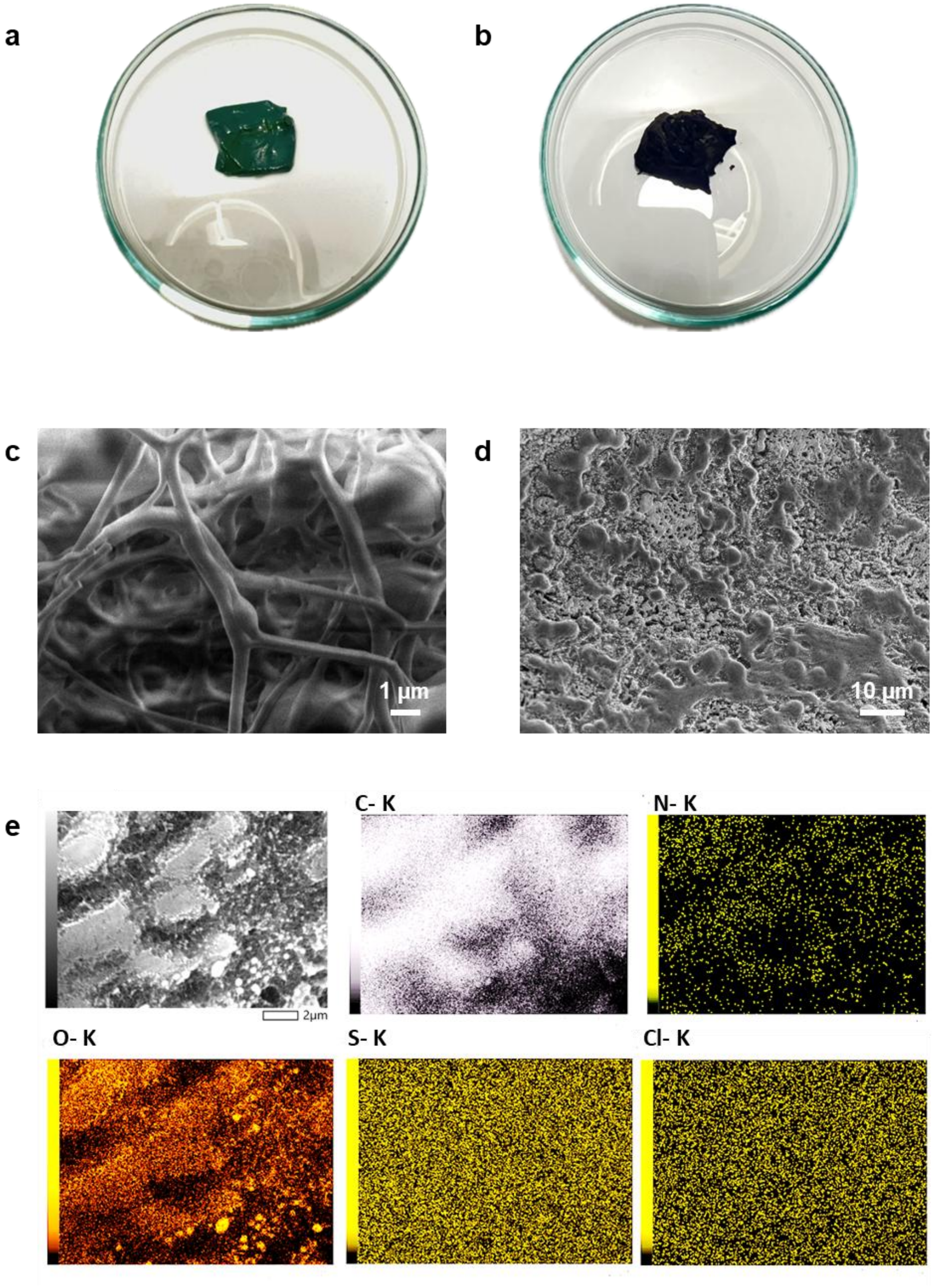


**Figure S2.** Digital image of spent fibers (SKL:CA 80:20) (a) before, (b) after carbonization, SEM images of (c) thermostabilized, (d) carbonized spent fibers and (e) EDS mapping of carbonized spent fibers (SKL:CA 80:20). Scale bar in the panel (e) is same for all the EDS mapping given.

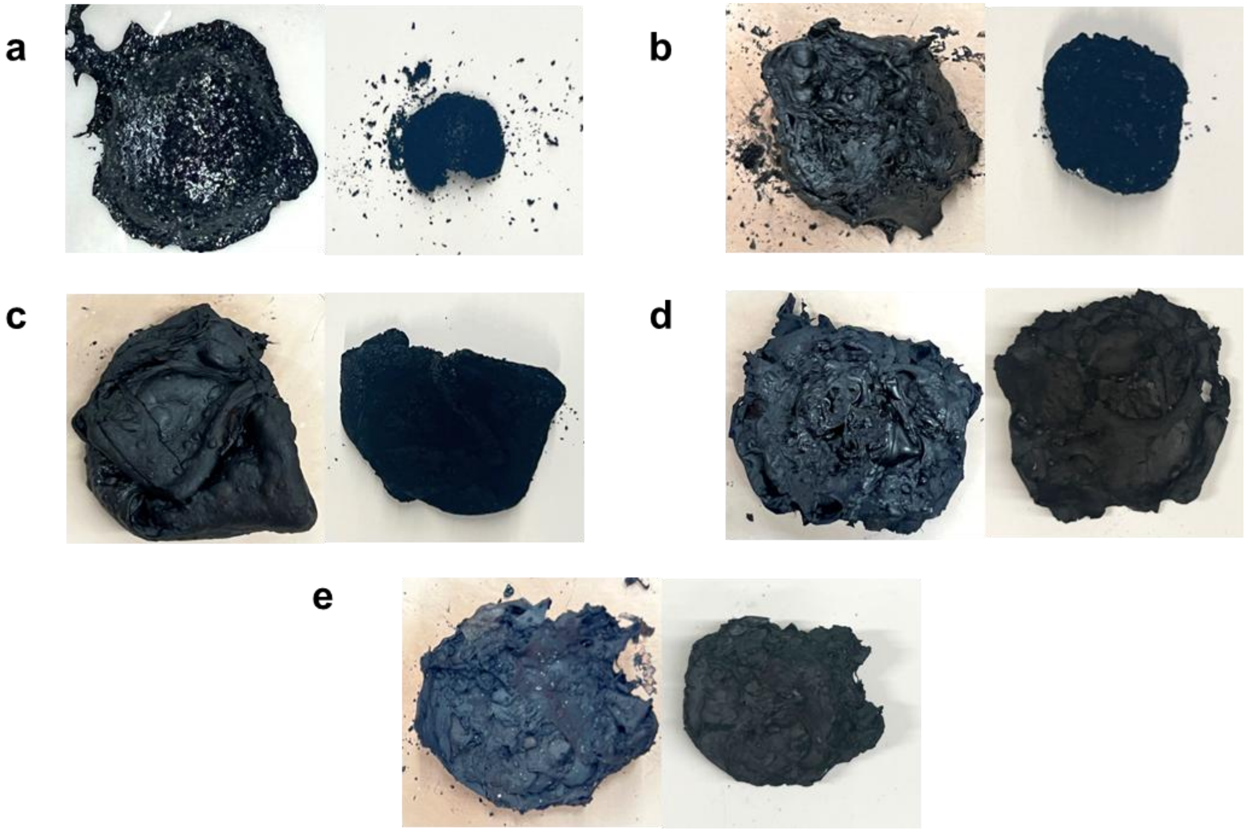


**Figure S3.** Digital images of the thermostabilized (left) and carbonized (right) SKL–CA nanofibers with composition in wt.% (a) 0:100, (b) 50:50, (c) 60:40, (d) 70:30 and (e) 80:20 respectively.

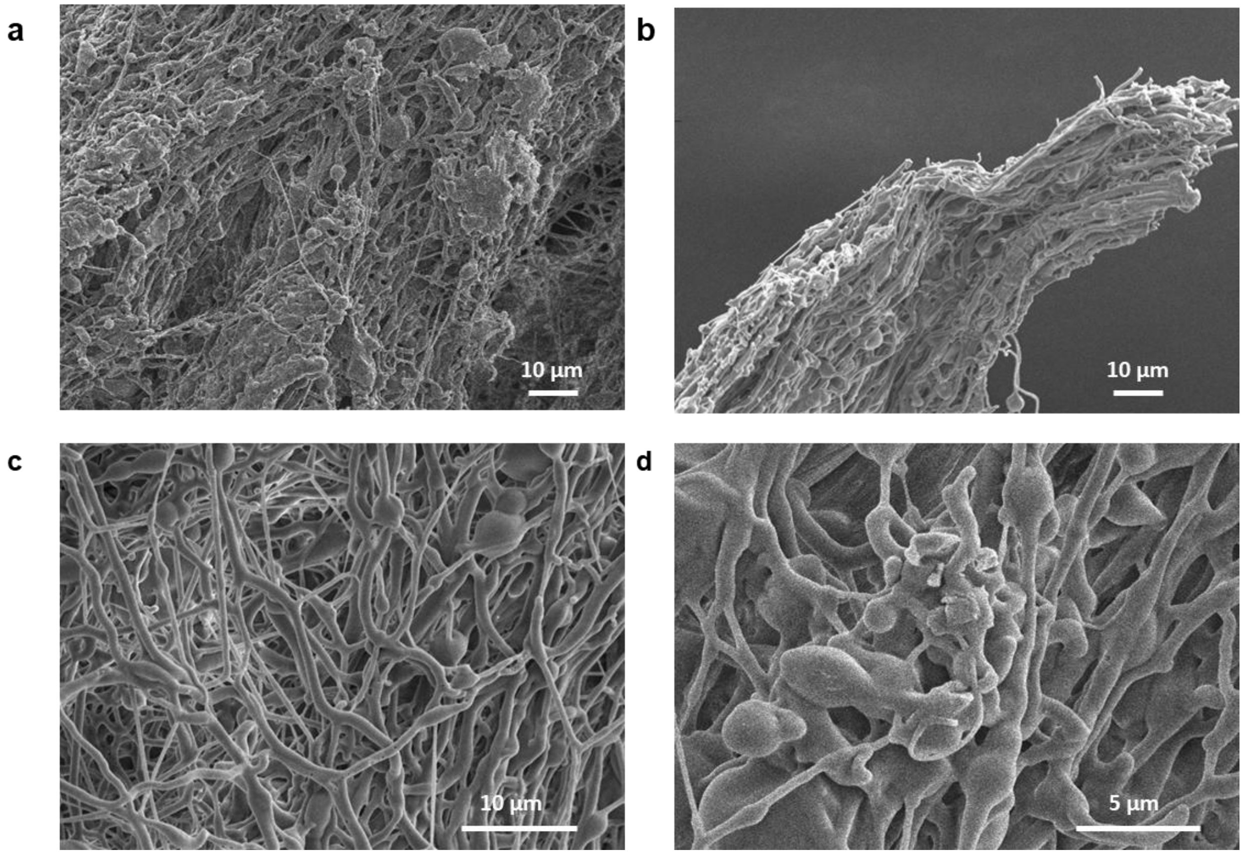


**Figure S4.** Scanning electron microscopic images of thermostabilized SKL–CA nanofibers with composition (a)–(d) 70:30 wt.%. at different magnifications.

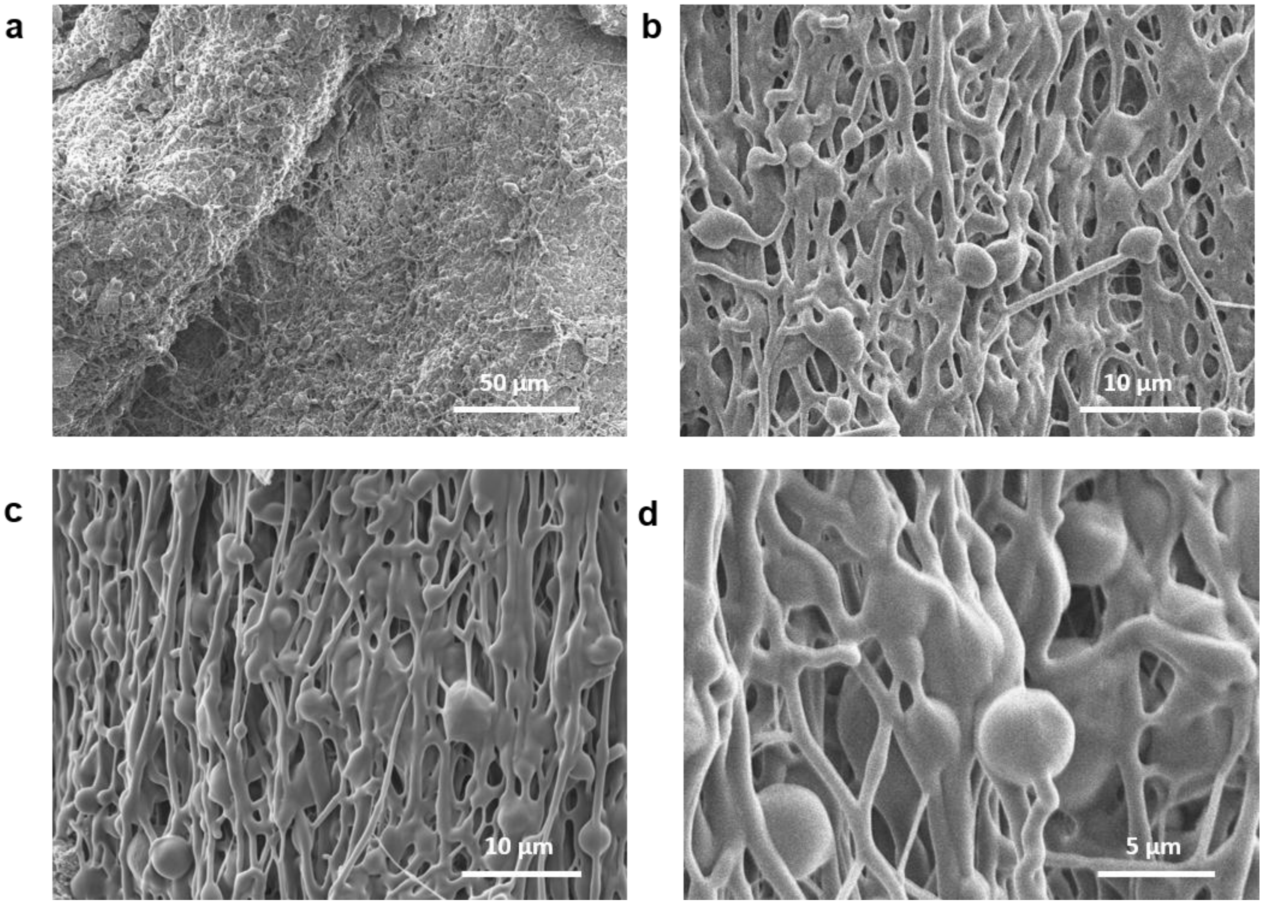


**Figure S5.** Scanning electron microscopic images of thermostabilized SKL–CA nanofibers with composition (a)–(d) 80:20 wt.%. at different magnifications.

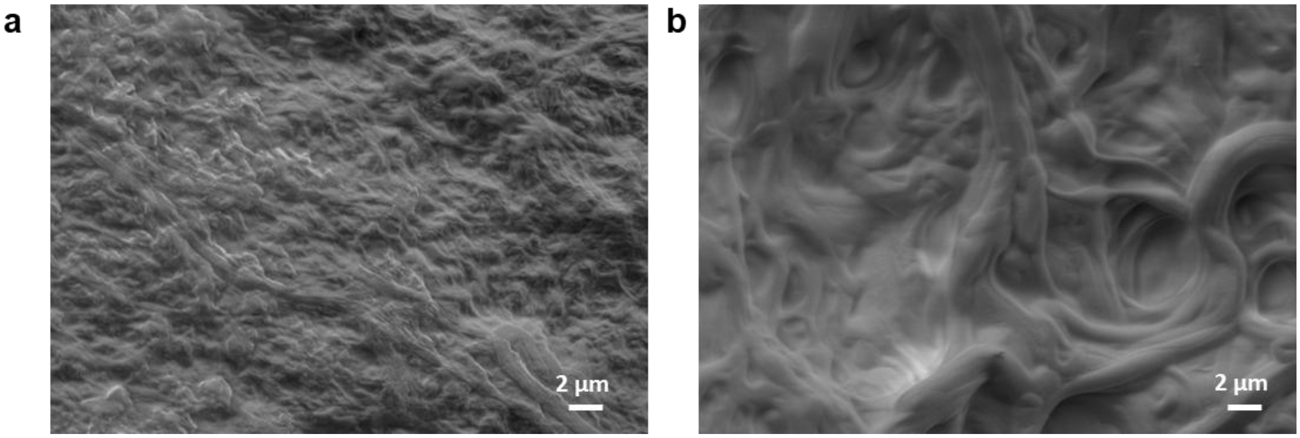


**Figure S6.** Scanning electron microscopic images of thermostabilized SKL–CA nanofibers with composition (a) 50:50 wt.% and (b) 60:40 wt.%.

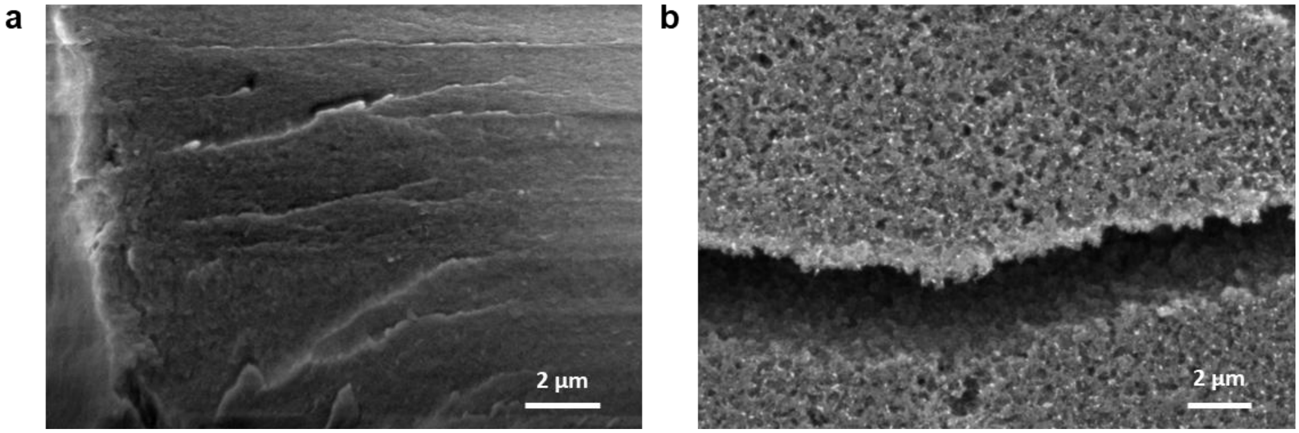


**Figure S7.** Scanning electron microscopic images of thermostabilized (a) and (b) carbonized CA nanofibers (SKL 0).

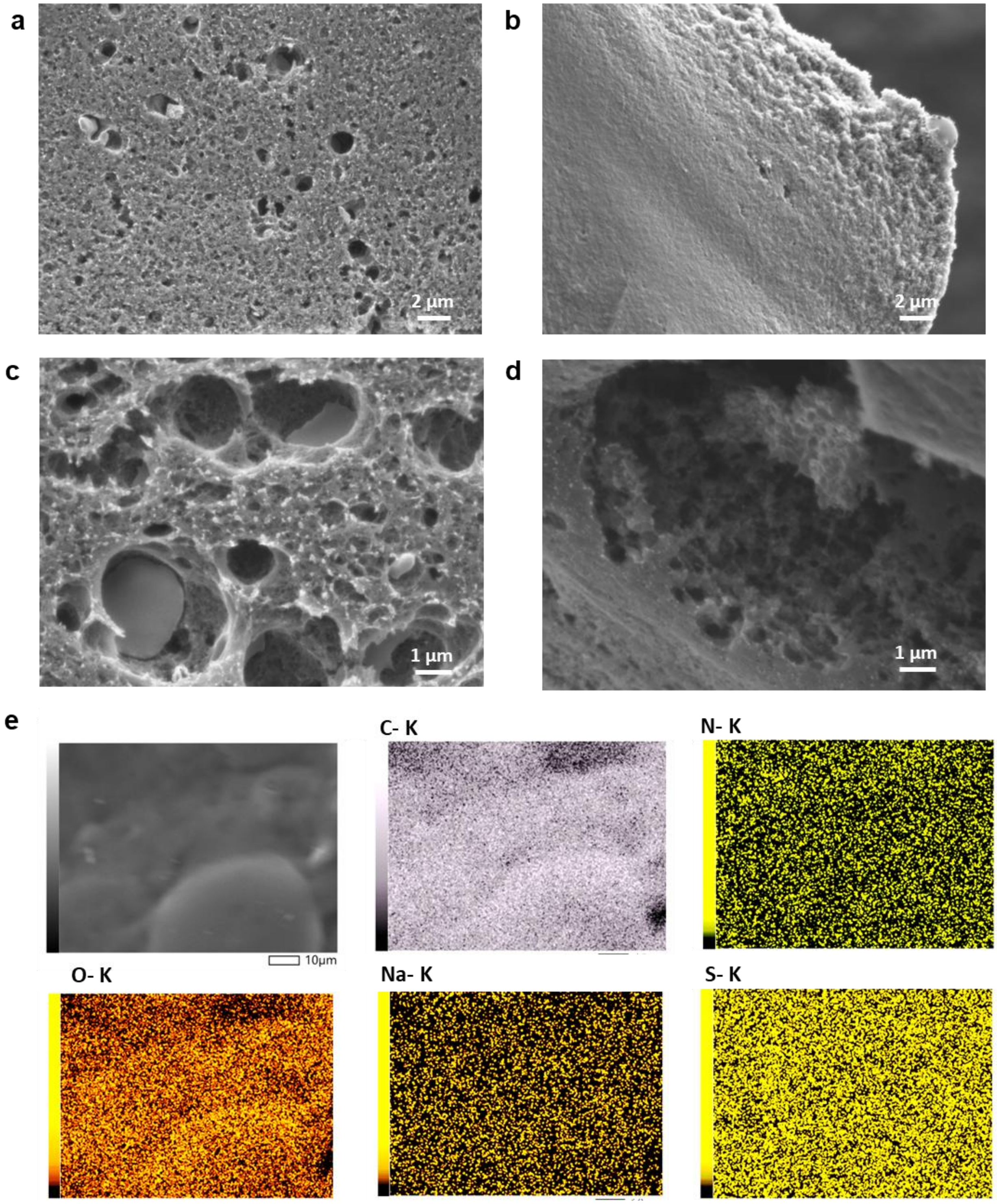


**Figure S8.** (a)–(d) Additional Scanning electron microscopic images and (e) EDS Mapping of carbonized SKL–CA nanofibers with 50:50 composition in wt.% (SKL 50). Scale bar in the panel (e) is same for all the EDS mapping given.

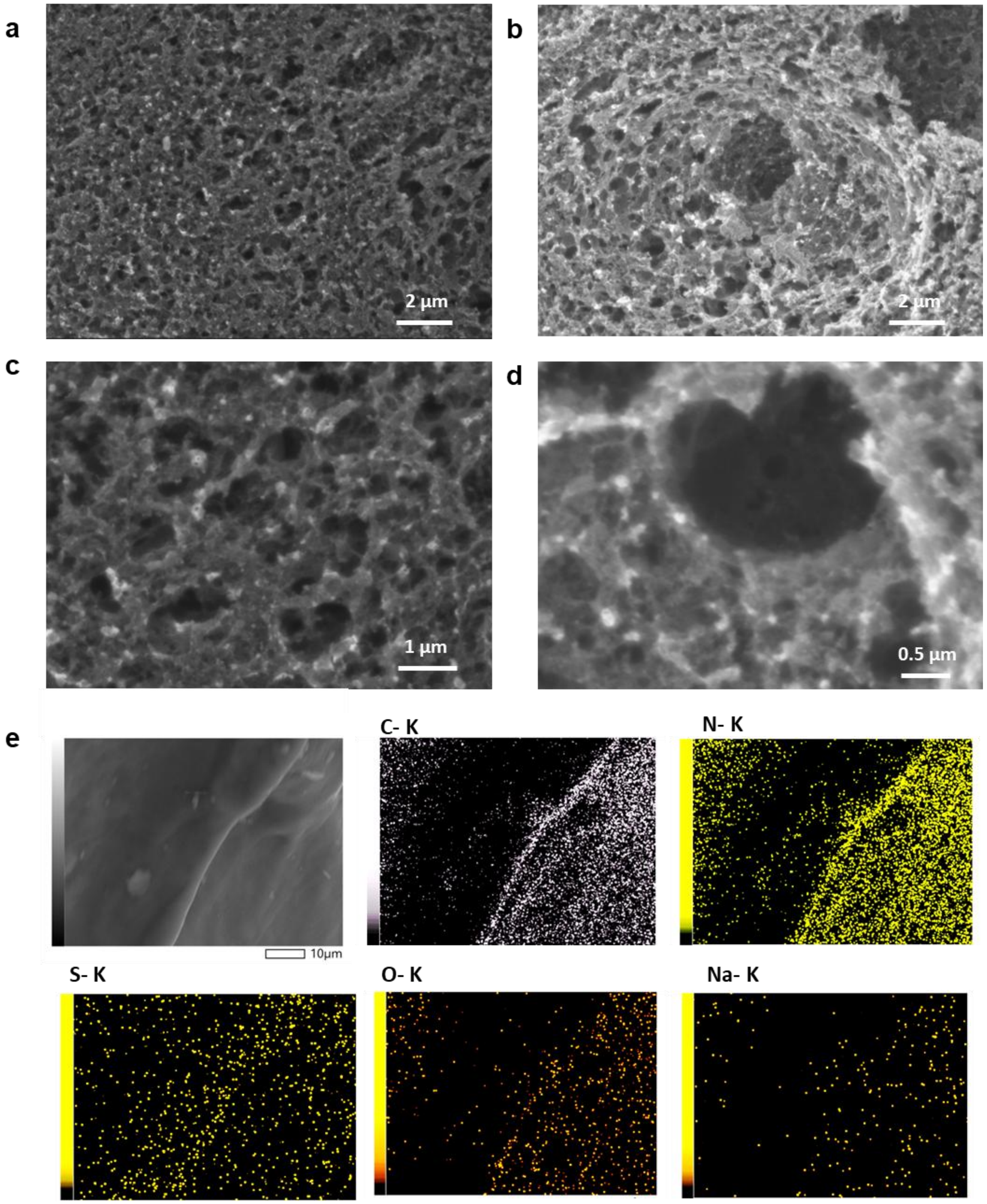


**Figure S9.** (a)–(d) Additional Scanning electron microscopic images and (e) EDS Mapping of carbonized SKL–CA nanofibers with 60:40 composition in wt.% (SKL 60). Scale bar in the panel (e) is same for all the EDS mapping given.

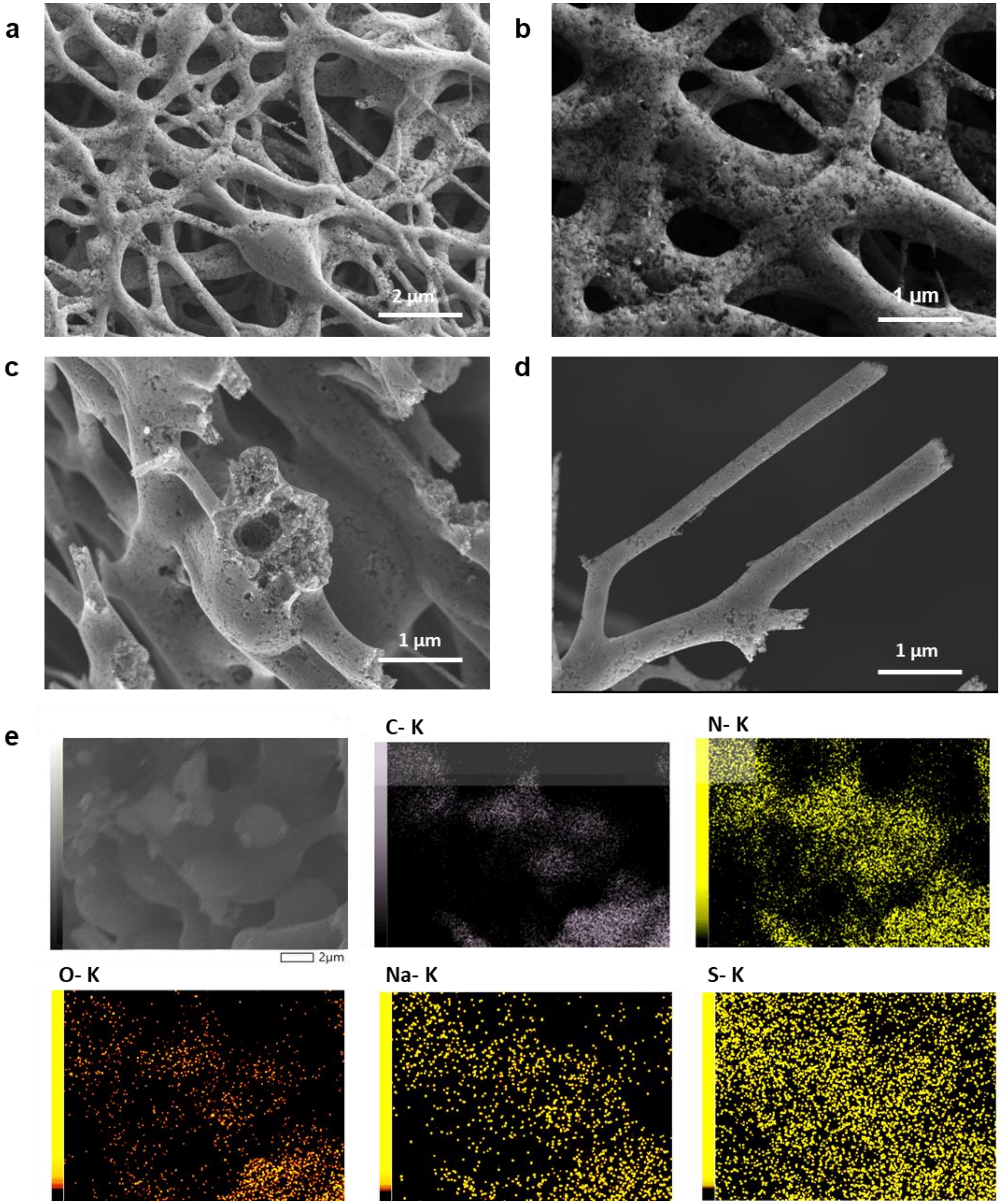


**Figure S10.** (a)–(d) Additional Scanning electron microscopic images and (e) EDS Mapping of carbonized SKL–CA nanofibers with 70:30 composition in wt.% (SKL 70). Scale bar in the panel (e) is same for all the EDS mapping given.

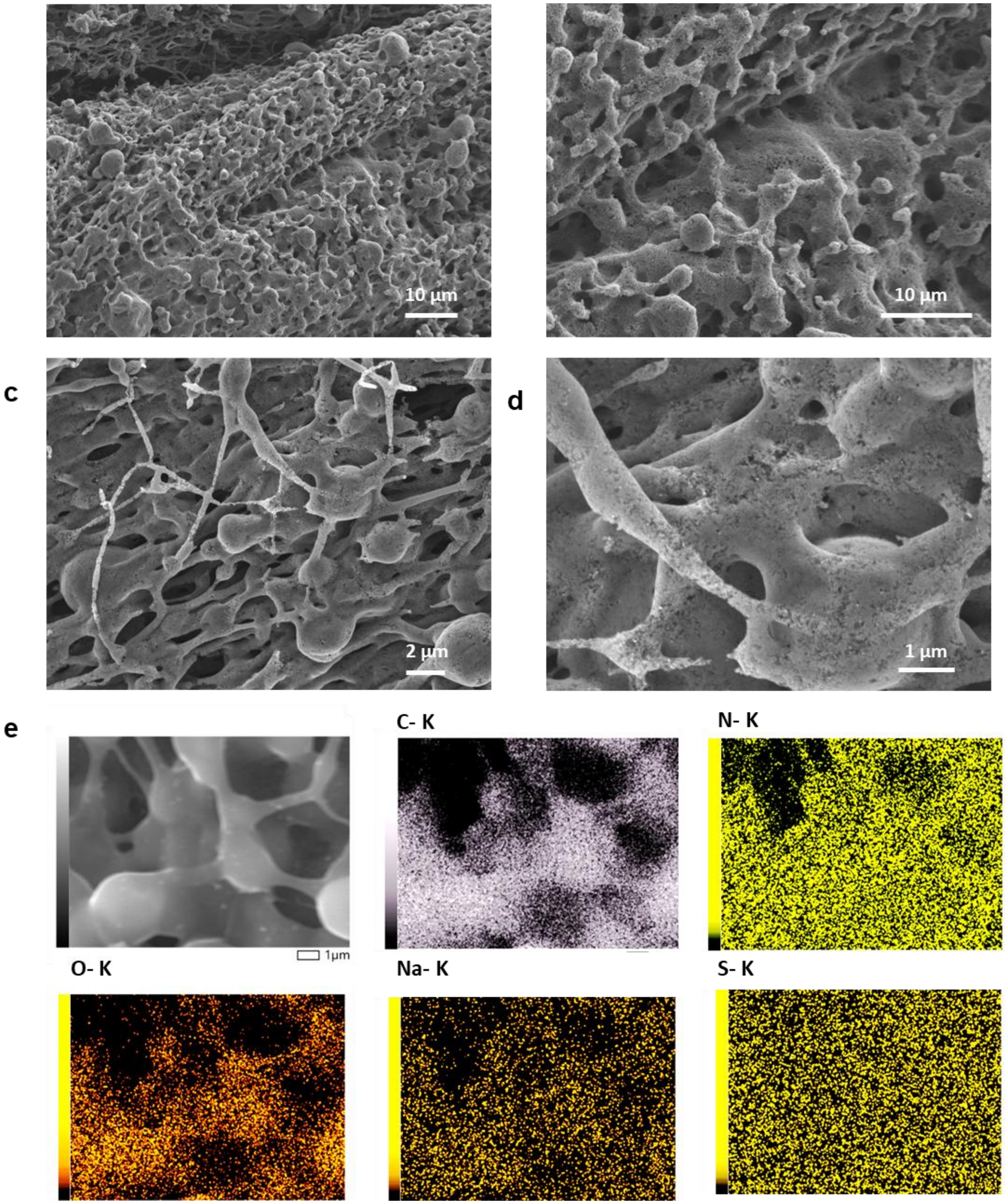


**Figure S11.** (a)–(d) Additional Scanning electron microscopic images and (e) EDS Mapping of carbonized SKL–CA nanofibers with 80:20 composition in wt.% (SKL 80). Scale bar in the panel (e) is same for all the EDS mapping given.

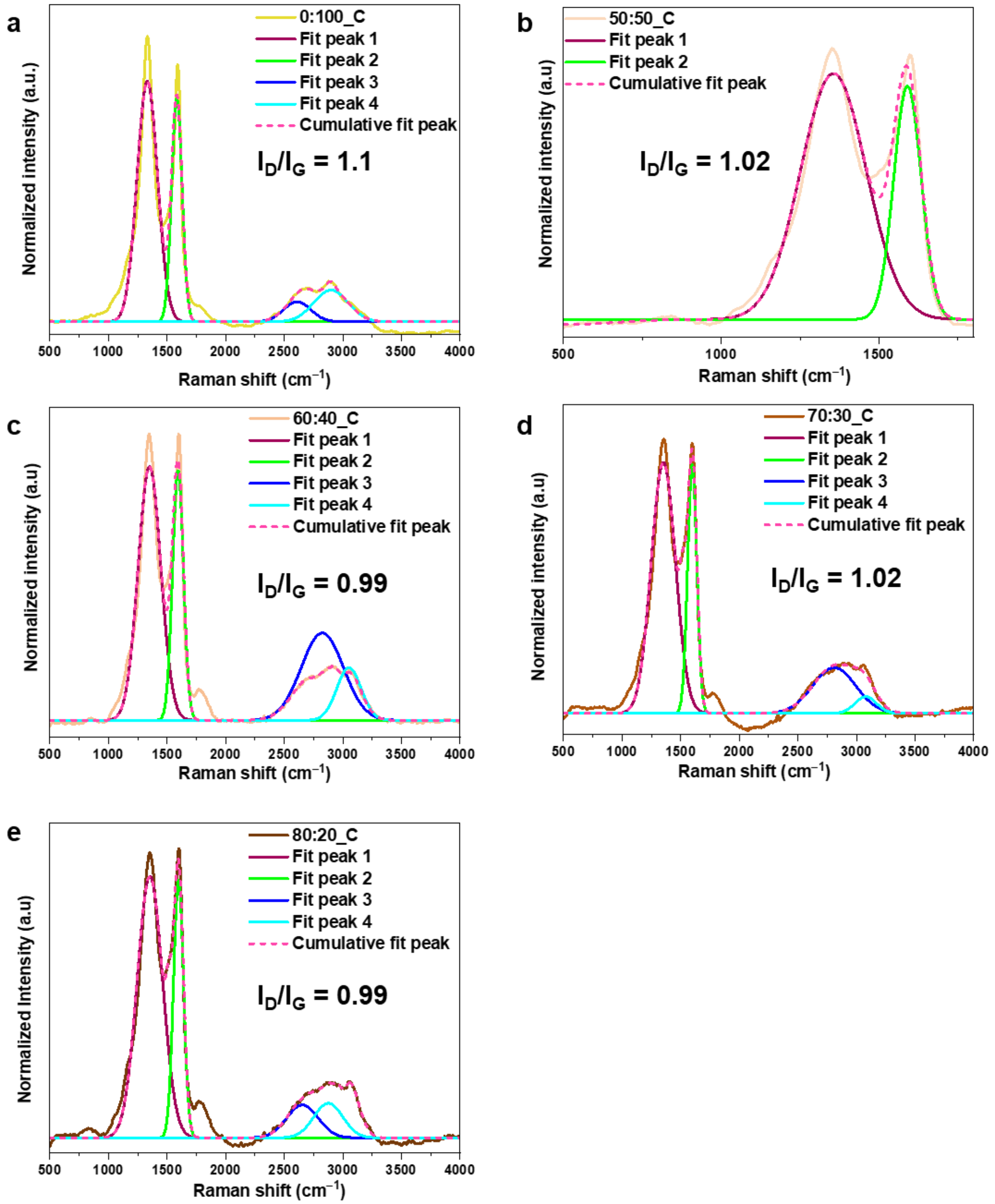


**Figure S12.** (a)–(e) Deconvoluted Raman spectra with Gaussian fit corresponding to **Fig. 7b**.

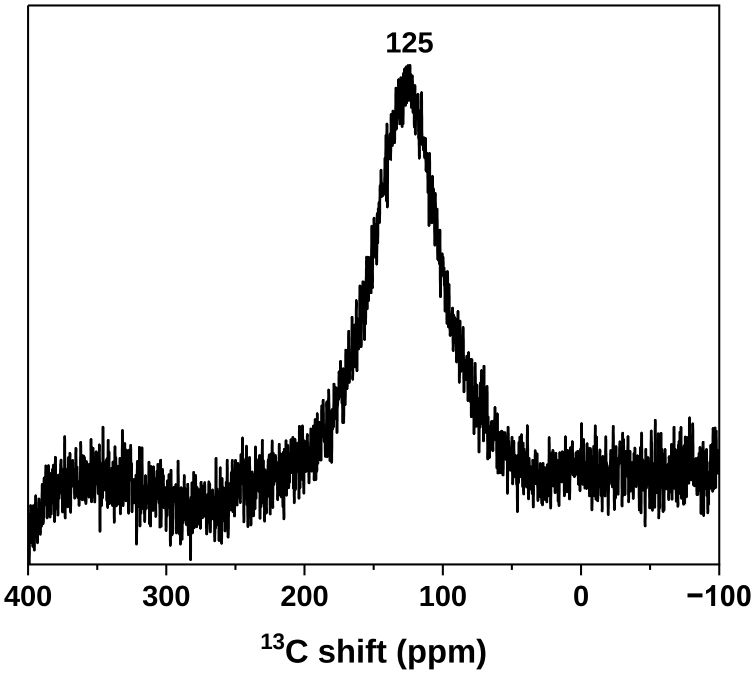


**Figure S13.** Solid-state $^{13}C$ MAS spectrum of carbonized SKL–CA nanofibers with 80:20 composition in wt.% (SKL 80).

**References:**


1. Cao, W. *et al.* Novel post-treatment of ultrasound assisting with acid washing enhance lignin-based biochar for CO2 capture : Adsorption performance and mechanism. *Chem. Eng. J.* **471**, 144523 (2023).

2. Pan, Z. *et al.* Lignin-based hierarchical porous biochar prepared from negative pressure pyrolysis enhanced CO2 and VOCs adsorption. *Sep. Purif. Technol.* **345**, 127398 (2024).

3. Sun, Y., Yang, G., Zhang, J., Wang, Y. & Yao, M. Activated Carbon Preparation from Lignin by H3PO4 Activation and Its Application to Gas Separation. *Chem. Eng. Technol.* **35**, 309–316 (2012).

4. Chen, W., Wang, X., Hashisho, Z., Feizbakhshan, M. & Shariaty, P. Template-free and fast one-step synthesis from enzymatic hydrolysis lignin to hierarchical porous carbon for CO2 capture. *Microporous Mesoporous Mater.* **280**, 57–65 (2019).

5. Zhao, B. *et al.* Lignin-Based Porous Supraparticles for Carbon Capture. *ACS Nano* **15**, 6774–6786 (2021).

6. Saha, D. *et al.* CO2 capture in lignin-derived and nitrogen-doped hierarchical porous carbons. *Carbon N. Y.* **121**, 257–266 (2017).

7. Liu, D. *et al.* Insights into mechanochemical assisted preparation of lignin-based N-doped porous carbon with tunable porosity and ultrahigh surface oxygen content for efficient CO2 capture. *Sep. Purif. Technol.* **347**, 127657 (2024).

8. Tkachenko, O. *et al.* Kraft Lignin-Derived Microporous Nitrogen-Doped Carbon Adsorbent for Air and Water Purification. *ACS Appl. Mater. Interfaces* **16**, 3427–3441 (2024).
